# Prescription charge policy acceptance among UK adults with and without long-term health conditions: a mixed-method survey

**DOI:** 10.1136/bmjopen-2024-085345

**Published:** 2024-09-24

**Authors:** Megan Rose Readman, Megan Polden, Lisa Brighton, Ayomide Oluseye, Ian Fairman, Ian Parkinson, Caroline Parkinson, Clarissa Giebel

**Affiliations:** 1 Department of Primary Care and Mental Health, University of Liverpool, Liverpool, UK; 2 Department of Psychology, Lancaster University, Lancaster, UK; 3 National Institute of Health Research Applied Research Collaboration North West Coast, Liverpool, UK; 4 Division of Health Research, Lancaster University, Lancaster, UK; 5 Department of Palliative Care, Policy, and Rehabilitation, King's College London, Cicely Saunders Institute, London, UK; 6 Faculty of Wellbeing, Education and Language Studies, The Open University, Milton Keynes, UK

**Keywords:** prescriptions, public health, health policy

## Abstract

**Abstract:**

**Objectives:**

Since their introduction in 1952, per-prescribed item charges in England have continually risen. This study investigated the acceptability and impact of per-prescribed item charges, and awareness and use of initiatives designed to reduce prescription charge financial burden (the prescription prepayment certificate (PPC) initiative), in people living with and without long-term health conditions (LTHCs) in the UK.

**Design:**

Cross-sectional mixed-method survey of people with and without an LTHC across the UK.

**Participants:**

381 people, 267 people with an LTHC and 114 people without an LTHC, participated.

**Outcome measures:**

Acceptability and impact of prescription charge policy, awareness and use of the PPC.

**Results:**

Over half (53.2 %) of participants disagreed with current per-prescribed item charges. In most domains, the impact of prescription charges did not differ between people with and without LTHCs. However, people with LTHCs were more likely to report financial burden and deviate from prescribed medication regimes. 35.29% of respondents were aware of the PPC, with people with LTHCs being more likely to be aware of and use this initiative. Qualitative findings indicate perceived inequalities in current policy with themes including (1) the need for re-evaluation; (2) the burden of prescription charges; (3) inconsistencies and inequalities in current policy; and (4) positive reflections of prescription charge policy.

**Conclusions:**

Inconsistencies in current policy and a lack of public support may suggest that a re-evaluation of current policy is required. The lack of difference in the impact of prescription charge policy between people with and without LTHCs indicates that the effects of such policy are not constrained to people with LTHCs. Thus, policy amendments would benefit the wider population. Systematic efforts to increase awareness of the PPC and reduce inequalities in medical exemption criteria are suggested.

**Trial registration number:**

Study protocol and analysis strategy are preregistered on Open Science Framework (https://shorturl.at/IrvnS).

**STRENGTHS AND LIMITATIONS OF THIS STUDY**

- This study included free-text qualitative response options, thereby allowing participants to express their opinions beyond the confines of Likert scale responses.
- The study sample had a greater proportion of females, people from white ethnic groups and people with high educational attainment; therefore, caution should be applied when generalising these findings to the rest of the UK population.
- While this is an England-centric study, our findings may have important implications for other countries who are currently considering alterations to prescription charge policy (eg, The national pharmacare policy of Canada).
- The current study did not examine the impact of polypharmacy and multimorbidity; therefore, conclusions cannot be drawn on the impact of the level of reliance of prescription charges on individual’s perceptions.

## Background

 Prescription charges, an upfront monetary fee associated with prescribed medications, were first introduced in the UK in 1952.^1^ Excluding the period between 1965 and 1968 when prescription charges were abolished in the UK, the price per prescribed item has continually risen, with the latest charge of £9.90 per prescribed item being brought into effect in April 2024.^1^ In 1968, a system of exemptions from prescription charges was introduced. This system enables people meeting the following criteria to be exempt from paying per prescribed item charges: (1) people in defined age groups (people under the age of 16, people aged 16–18 in full-time education and people over the age of 60), (2) people who are pregnant or have had a baby in the last 12 months, (3) people who receive certain state benefits and (4) people living with *specific* medical conditions (MedEx criteria). Subsequently, people aged 18–60, who do not receive state benefits, are not pregnant or have had a baby in the last 12 months, and are not living with medical condition covered by the MedEx criteria, are required to pay an upfront fee of £9.90 per prescribed medication on collection. For the financial period of 2021–2022, it was estimated that £652 million revenue was collected from prescription charges in the UK.^2^

**Figure 1:**
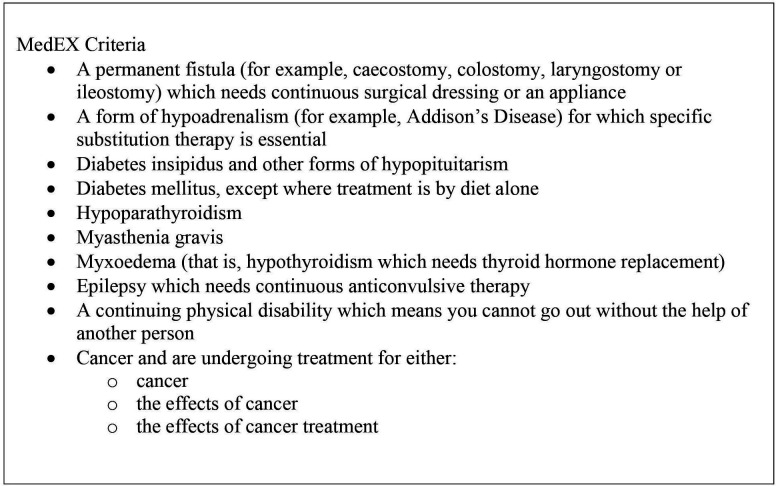
Current MedEx criteria.

The exemption from paying per prescribed item charges may relieve financial burden and benefit people who fall within one of the eligible categories. However, this system of exemption has only been updated once since its conception, with cancer being added to the MedEx criteria in 2009.^3^ Presently, only 10 specific medical conditions are covered under the MedEx criteria (see figure 1 for full MedEx criteria). There are, however, many more long-term health conditions (LTHCs), including, but not limited to, neurological conditions (e.g., Parkinson’s disease, motor neuron disease, multiple sclerosis and dementias), psychiatric conditions (e.g., depression, anxiety, bipolar disorder and schizophrenia), autoimmune conditions (e.g., lupus and Sjögrens syndrome), respiratory conditions (e.g., asthma, chronic obstructive pulmonary disease and cystic fibrosis) and blood disorders (e.g, anaemia and haemophilia), which require prolonged pharmacological treatment that is not included on this exemption list.

In an attempt to mitigate the financial burden of prescription charges, particularly for people living with LTHCs, the Department of Health and Social Care introduced a Prescription Prepayment Certificate (PPC). PPCs serve to limit the amount people pay annually for prescriptions by enabling individuals to make a one-off payment that covers all prescription charges for the following year/3-month period.^3^ PPCs specifically aim to reduce the costs of prescriptions for people who repeatedly require multiple prescribed items, with the PPC minimising prescription costs for people who buy more than 3 prescribed items in 3 months or 12 prescribed items in 12 months. Theoretically, this initiative will substantially reduce financial burden for people living with LTHCs. However, the 2023 Prescription Charge Coalition survey^4^ observed that while overall awareness of the PPC among people living with LTHCs is high, many people are not made aware of the PPC for a minimum of 6 months following diagnosis (38% not being made aware for over 1 year; 15% not being made aware for between 6 months to 1 year). Furthermore, awareness of the PPC appears to be driven by word of mouth with 31% of respondents citing the source of awareness being friends/family. Thus, the accessibility and effectiveness of this initiative may be questioned.^4^

Analyses of the financial impact of prescription charges, particularly on people with long-term conditions, are limited. However, some research suggests that prescription charges may act as a barrier to the use of prescribed medication and result in negative impacts on health outcomes due to deviations from medication regimes.^5^ For example, the 2014 Prescription Charges Coalition survey found that 37% of people with long-term conditions had deviated from their medication regime or inhibited taking medication due to the cost.^6^ Of those people, 75% reported that this negatively impacted their health with 10% resulting in a hospital admission after failing to take prescribed medication.^6^

The present cross-sectional mixed-methods survey aimed to (1) further our understanding of the acceptability of current UK prescription charge policy and the impact of prescription charges on both people living with and without LTHCs, and (2) ascertain the level of awareness of current initiatives to reduce the financial burden of per-prescribed item charges. To do so, both people living with and without LTHCs shared their opinions and experiences regarding the current per-prescribed item charges, the impact of prescription charges and engagement with the PPC. We hypothesised that, compared with individuals without LTHCs, individuals who live with an LTHC will be substantially more opposed or less likely to support the prescription charge policy. Moreover, we hypothesised that individuals living with LTHC would report greater impact of per-prescribed item charges than people without LTHCs. Finally, based on the findings of the 2023 Prescription Charge Coalition survey,^4^ we hypothesised that overall awareness of the PPC would be low.

## Methods

This study’s protocol, planned statistical analyses and data analysis code book can be found on the Open Science Framework (OSF; https://shorturl.at/IrvnS). The only minor deviation from the protocol was the number of participants who had an LTHC compared with those without. Specifically, we intended to recruit equal numbers of people with and without LTHCs. However, due to the application of opportunity sampling 70% of participants were classified as living with an LTHC and 30% were classified as not living with an LTHC. All data analysed in this study are publicly available on the OSF project.^7^

### Patient and public involvement

Three people (two people living with an LTHC and one family member of a person with an LTHC) were involved in the design, reporting and dissemination plans of this research. Please refer to the Materials (Survey development) section for further details.

### Participants

Participants were recruited via social media advertisements, including adverts issued by Parkinson’s UK, and email adverts to local research networks. Thus, participation was self-selected. A minimum sample size of n=380 was calculated using G*Power (3.1.9.7)^8^ (see protocol (online supplemental material) for full sample size justification). Participants were eligible if they were aged ≥18 years and lived in the UK. As the survey asked participants to reflect both on current and previous experiences, both participants who currently pay for their prescriptions and those who do not were eligible for inclusion.

## Materials

### Survey development

The survey was codeveloped with the project public advisory team which consisted of two people living with LTHCs (IF and CP) and family member of a person living with an LTHC (IP). Codevelopment occurred following an five steps process: (1) The Prescription Charge Coalition 2023 survey was consulted and the gaps in this survey assessed (MRR and MP), (2) a skeleton survey was developed based on the gap identified in the Prescription Charge Coalition 2023 survey and existing literature (MRR and MP), (3) the wider research team provided feedback on the skeleton survey and it was revised accordingly, (4) public advisors provided feedback on the skeleton survey, particularly focusing on whether they feel it appropriate addressed their lived experience, and it was revised accordingly, (5) the amended survey was redistributed to public advisors for assessment of readability and accessibility. The resulting survey was then piloted on seven participants (three people with LTHCs and four people without LTHCs). The pilot revealed that the survey was appropriate in terms of readability, length and content and so was finalised.

#### Survey measures

##### Demographics

Demographic variables included self-identified gender, ethnicity, current employment status, highest level of educational attainment, current household income before taxes and deductions, the number of people residing in the same household aged >14 years and the number of people residing in the same household aged <14 years (see online supplemental table S1). The first three digits of the respondent’s postcode were collected to generate an Index of Multiple Deprivation (IMD) quintile. IMD is an index of neighbourhood deprivation generating one deprivation score for income, employment, education, health, crime, barriers to housing and services and living environment.^9^ In accordance with the Ministry of Housing, Communities & Local Government English indices of deprivation 2019, quintile ‘1’ indicates the most deprived neighbourhoods, and quintile ‘5’ indicating the least deprived neighbourhoods.^8^ IMD values, for each participant, were obtained by inputting postcode data into the Ministry of Housing, Communities & Local Government English indices of deprivation 2019 Postcode Lookup (https://imd-by-postcode.opendatacommunities.org/imd/2019).

##### Health background

Participants were asked to disclose whether they are currently living with any of 12 preset LTHCs, including other non-specified LTHC. The preset list of LTHCs was developed based on the prespecified conditions included within the Prescription Charges Coalition 2023 survey^4^ and was updated to ensure both and the National Health Service (NHS) Quality and Outcomes Framework 20 common conditions^10^ and UK Department of Health and Social Care Major Conditions Strategy major condition groups^11^ were included. The inclusion of the other non-specified group further ensured that people who identified with all LTHCs were included. Participants who indicated they have an LTHC were further prompted to disclose the nature of this condition(s) (see online supplemental table S2 for full questions).

##### Prescription medication taking behaviour

Participants were asked to disclose whether they are prescribed, and taking, long-term medications, the quantity of medications, whether they pay for medications and the monthly costs associated with these medications (see online supplemental table S3).

##### Awareness and use of current initiatives to reduce financial burden (PPC)

To ascertain awareness and use of the PPC initiative, participants were asked to whether they were aware of (“Have you ever heard of an NHS Prescription Prepayment Certificate (PPC)?”), and have previously or currently use PPC initiative (“Do you currently have an NHS Prescription Prepayment Certificate (PPC)” and “If/When you were required to pay for your prescriptions did you have an NHS Prescription Prepayment Certificate (PPC)?”). To ensure that differences in the terminology used to refer to the PPC did not impact participants responses, this question was accompanied with a short description of what the PPC (“A certificate that covers all your NHS prescriptions for a set price, like a prescription charge season ticket”) is and a link to the NHS website for further clarification of what the PPC initiative is. Participants who indicated that they were aware of the PPC were asked where they had about it, and why they had chosen to use (or not use) this initiative (see online supplemental table S4).

##### Acceptability and impact of prescription charge policy

Participants were asked to rate agreement, on a five-point Likert scale ranging from strongly disagree to strongly agree, to five statements regarding current prescription charge policy (“To what extent do you agree with the below statement; (1) The current price of prescriptions is fair and reasonable, (2) I currently or have previously considered the price of the prescription before collecting it, (3) I currently/ previously have had to make financial sacrifices to afford my prescriptions, (4) The costs of my prescriptions currently places or has previously placed additional financial burden on me and/or my family, (5) The costs associated with my medications currently places or has previously placed emotional stress on myself and/or my family”) . An additional two binary response questions, with follow-up five-point Likert scale questions, were included to assess the impact of per-prescribed item charges on adherence to prescribed medication regimes: (1) Have you previously (even just once) elected not to collect a prescription due to the costs associated with it?, (2) Have you ever (even just once) altered or deviated from your medication regime due to cost that is, halving medication dose etc?. A free-text response question was also included to allow participants to qualitatively express their opinions on these policies further (see online supplemental table S5).

### Procedure

The survey was completed online only. All data were collected between August and December 2023. On following the online survey link, participants were directed to the study information sheet clearly detailing the aims of the study and associated task. If participants elected to proceeded, they then provided digitalised informed consent and completed a reCAPTCHA identification check to ensure no bots completed the study. On attainment of informed consent, participants were presented the main survey. All participants provided consent for their anonymised date to be included in the present publication and other outputs relating to this study.

#### Data analysis

Descriptive statistics were used to describe the population and compare the levels (%) of acceptability/support for the policy across all participants as one group, and between participants living with and without LTHC. Multinomial logistic regressions, controlling for age, gender, ethnicity, education, household income and IMD, were used to examine whether participants with an LTHC (vs without) were more likely to agree or disagree with current policy and experience negative impacts. Binary logistic regressions, controlling for age, gender, ethnicity, education, household income and IMD, were used to investigate the impact of per-prescribed item charges on prescription medication adherence and the awareness and use of the PPC initiative. Multinomial logistic regression results are presented as relative risk ratio (RRR) along with 95% CIs and p value. Binary logistic regression results are presented as ORs along with 95% CIs and p value.

To maintain participants right to not provide data for a given question, the survey was designed in a way which enabled response submission with incomplete answers. Therefore, for all analyses, the entire sample who have data available for the given outcome variable will be analysed. To maximise the quantity of data available, participants with missing data, and participants who responded ‘prefer not to say’, were excluded from the analysis for which they had missing data only.

To account for multiple comparisons and reduce the likelihood of type I error, the Bonferroni correction^12 13^ was applied to all logistic regressions (both binary and multinomial). The resulting significance level of p <0.0045 (0.05/11) was applied. Full information regarding variable transformations can be found in the study protocol (online supplemental materials), and the accompanying raw data code can be found in online supplemental materials.

Additional exploratory analyses including only participants who are currently paying for their prescriptions are presented as online supplemental materials. Findings differed from the primary analyses only in relation to the experience of financial burden (see online supplemental tables S6 and S7 for full analysis and S8 for a summary of differences between the primary and exploratory analyses).

Qualitative free-text responses were analysed using inductive reflexive thematic analysis.^14^ All free-text responses were analysed by two research team members (MRHR and MP) to enhance interpretation. Free-text responses were first coded individually, with codes and emerging themes discussed jointly to generate overarching themes and subthemes.

## Results

### Sample characteristics

**Figure 2:**
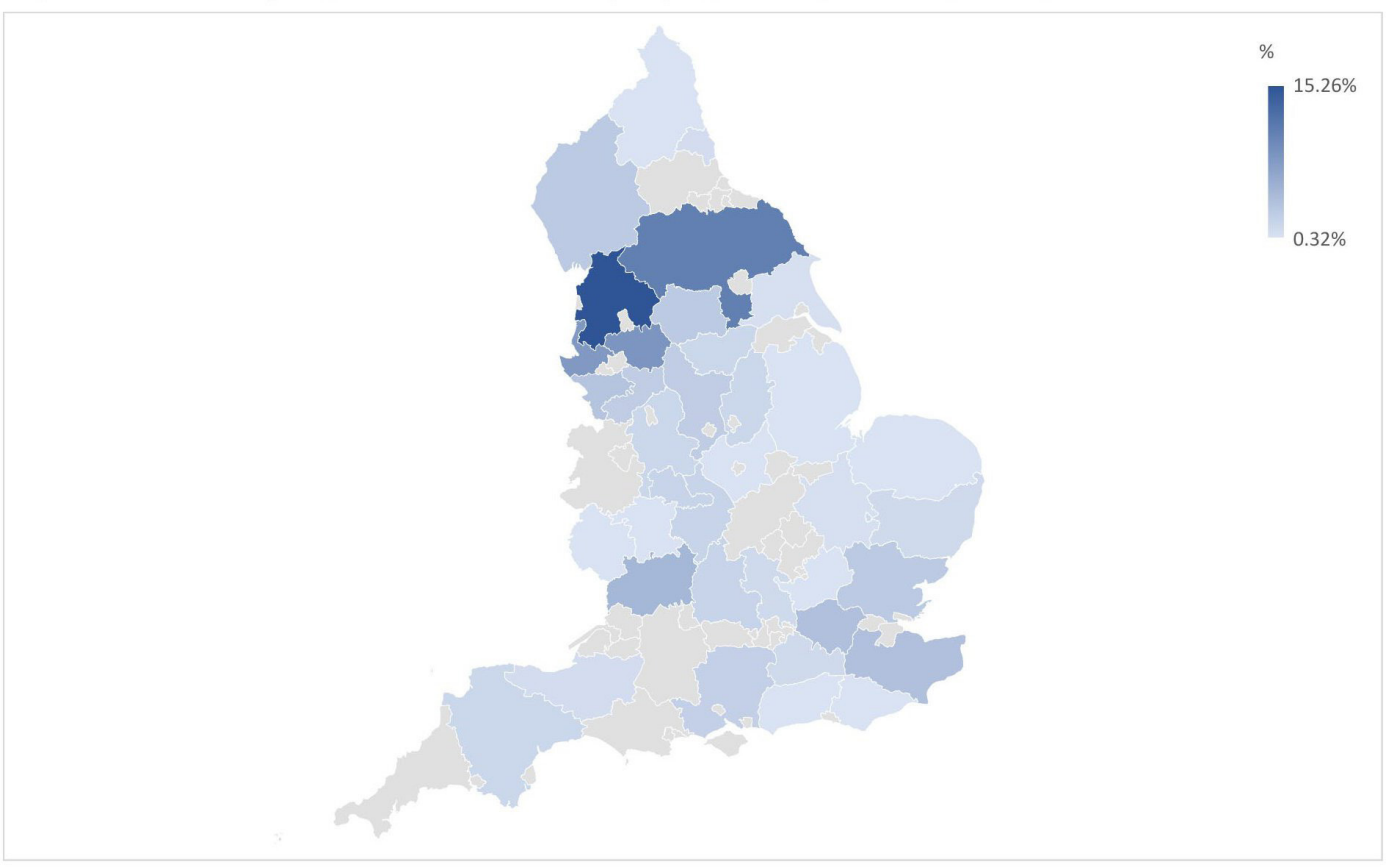
Heat map representation of the geographical spread of participants.

381 people aged 19–84 (*M*=47.29 (17.81)) participated in this survey: 267 people (70%) who reported having an LTHC and 114 people (30%) who did not report having an LTHC. Across the full sample, participants were predominantly female (n=256, 67.19%) from a white ethnic background (n=358, 93.96%), educated up to degree level or equivalent (n=277, 72.70%), and in work (working full (n=163, 42.78%), or part time (n=62, 16.27%)). Participants spread all IMD quintiles and homes of residence spanned most of England (see figure 2 for the local authority geographical regions represented; see table 1 for full sample characteristic breakdown).

**Table 1:** Sample demographics

	Participants with a long-term health condition (n=267)	Participants without a long-term health condition (n=114)	Total sample (n=381)	P, χ2*
N (%)				
Age (years) (Mean (SD))	50.86 (17.34)	39.13 (16.17)	47.29 (17.81)	t (201.94) = −5.94, p<0.001
Gender				χ2 (3) = 2.62, p=0.45
Female	174 (65.17)	82 (71.93)	256 (67.19)	
Male	90 (33.71)	32 (28.07)	122 (32.03)	
Non-binary / third gender	2 (.75)		2 (.52)	
Prefer not to say	1 (.37)		1 (.26)	
Ethnicity				χ2 (11) = 14.35, p=0.21†
White	252 (94.38)	106 (92.98)	358 (93.96)	
BAME	13 (4.87)	8 (7.02)	21 (5.52)	
Prefer not to say	2 (.75)		2 (.52)	
Employment status				χ2 (5) = 29.45, p<0.001
Working full time	102 (38.20)	61 (53.51)	163 (42.78)	
Working part time	39 (14.61)	23 (20.18)	62 (16.27)	
Student	9 (3.37)	11 (9.65)	20 (5.25)	
Out of work	5 (1.87)	2 (1.75)	7 (1.84)	
Retired	102 (38.20)	16 (14.04)	118 (30.97)	
Prefer not to say	10 (3.75)	1 (.88)	11 (2.89)	
IMD‡				t (193.93) = 2.25, p=0.03
1	24 (10.67)	7 (7.08)	31 (9.57)	
2	60 (26.67)	20 (20.20)	80 (24.69)	
3	59 (26.22)	20 (20.20)	79 (24.38)	
4	63 (28.00)	43 (43.43)	106 (32.72)	
5	19 (8.44)	9 (9.09)	28 (8.64)	
No postcode data provided	42	15	57	
Education				χ2 (4) = 7.84, p=0.10
Degree level or above	187 (70.04)	90 (78.95)	277 (72.70)	
AS, A levels or equivalent	32 (11.99)	16 (14.04)	48 (12.60)	
GCSEs or equivalent	41 (15.35)	7 (6.14)	48 (12.60)	
No qualifications	3 (1.12)		3 (.79)	
Prefer not to say	4 (1.50)	1 (.87)	5 (1.31)	
Annual household income before tax and deductions				χ2 (11) = 18.69, p=0.07
£0 - £10 000	5 (1.87)	5 (4.39)	10 (2.62)	
£10,001- £20 000	11 (4.12)	6 (5.26)	17 (4.46)	
£ 20,001 - £30 000	44 (16.48)	5 (4.39)	49 (12.86)	
£ 30,001 - £40 000	37 (13.86)	15 (13.16)	52 (13.65)	
£ 40,001 - £50 000	26 (9.74)	17 (14.91)	43 (11.29)	
£ 50,001 - £60 000	28 (10.49)	14 (12.28)	42 (11.02)	
£ 60,001 - £70 000	23 (8.61)	17 (14.91)	40 (10.50)	
£ 70,001 - £80 000	19 (7.12)	8 (7.02)	27 (7.09)	
£ 80,001 - £90 000	11 (4.12)	5 (4.39)	16 (4.20)	
£ 90,001 - £100 000	11 (4.12)	5 (4.39)	16 (4.20)	
£ 100,001+	16 (5.99)	8 (7.02)	24 (6.30)	
Prefer not to say	36 (13.48)	9 (7.88)	45 (11.81)	
Currently prescribed, and taking, any long term medications				χ2 (11) = 151.68, p<0.001
Yes	238 (89.14)	30 (26.32)	268 (70.34)	
No	26 (9.74)	84 (73.68)	110 (28.87)	
Prefer not to say	3 (1.12)	0	3 (.78)	
Number of prescribed medications per day (medication types) Mean (SD)	4.32 (2.95)	1.87 (1.63)	4.04 (2.93)	t (57.38) = - 6.89, p<0.001
Number of tablets taken per day Mean (SD)	9.71 (7.17)	3.23 (3.99)	8.98 (7.18)	t (54.617) = - 7.35, p<0.001

* The p values and χ2 documented here were obtained from independent sample t-tests and chi-squared test of independence which examined whether demographic characteristics significantly differ across participant groups.

† Within the raw survey data participants self-identified 12 ethnicities. Hence, why the degrees of freedom for the χ2 analysis is 11. However, due to the number of respondents selecting non-white ethnicities for ease of interpretation, here we categorised ethnicity as white and BAME.

‡ IMD is an index of neighbourhood deprivation generating one deprivation score for income, employment, education, health, crime, barriers to housing and services, and living environment. In accordance with the Ministry of Housing, Communities & Local Government English indices of deprivation 2019, quintile ‘1’ indicates the most deprived neighbourhoods, and quintile ‘5’ indicating the least deprived neighbourhoods [8]. Not all participants provided postcode data, therefore, IMD could not be calculated for all participants. The percentages computed for the IMD variable are true percentages of the participants for which an IMD value could be calculated.

BAMEBlack and Ethnic Minority EthnicitiesIMDIndex of Multiple Deprivation

Participants who reported having an LTHC tended to be older, were more likely to be working full time and were from more deprived IMD localities. Neurological conditions, psychiatric impairments and hypertension were the most common LTHCs reported (see online supplemental table S9 for full breakdown of LTHCs reported). Most participants, who reported having an LTHC, reported having one condition (n=147, 55.06%). However, a substantial proportion of participants reported living with several LTHCs (2 conditions, n=78, 29.21%), 3 conditions, n=31, 11.61%)). A minority (n=26, 9.74%) of participants with an LTHC indicated that they were not currently taking any long-term medications. These participants disclosed living with LTHCs for which non-pharmacological therapies are often first-line treatment.

### Awareness and use of current initiatives to reduce financial burden (PPC)

Just over one-third (35.29%) of all participants had never heard of the PPC, with people with LTHCs (vs without) being significantly more likely to have heard of the PPC than people (OR=4.92; 95% CI 2.62, 9.47, p<0.001). However, 25.38% of people with LTHC had never heard of the PPC. Friend/family/word of mouth (32.73%) and pharmacy were the most frequently cited sources of awareness (31.82%).

Only 26.56% of all participants previously/currently use the PPC, with people with an LTHC (vs without) being more likely to currently/previously have used the PPC (OR=11.9; 95% CI 4.30, 43.1, p<0.001).

### Acceptability of per prescribed item charge policy

Across the total sample, most (53.21%) respondents disagreed with the current per-prescribed item charge, with participants indicating that they would be willing to pay between free of charge to a maximum of £8 (*M* =£3.49 (£2.33)) per prescribed item. There was no difference in acceptability of the current per-prescribed item charge between people with an LTHC and without (RRR=0.88; 95% CI 0.39, 1.96, p=0.7; see table 2).

**Table 2:** Multinomial logistic regression analyses examining differences in the acceptability of prescription charge policy and the impact of prescription charges between participants with and without LTHCs

					Multinomial logistic regression results (LTHC v No LTHC)		
	n	Agree (n(%))*	Neutral (n(%))*	Disagree (n(%))*	Relative risk ratio (RRR)	95% CI	p
Acceptability of per prescribed item charge policy							
To what extent do you agree with the below statement: The current price per prescribed item (£9.65) is fair and reasonable							
Total Sample	374	96 (25.67)	79 (21.12)	199 (53.21)	0.88	0.39, 1.96	0.70
LTHC	264	63 (23.86)	61 (23.11)	140 (53.03)			
No LTHC	110	33 (30.00)	18 (16.36)	59 (53.64)			
Impact of prescription charges on behaviours							
To what extent do you agree with the below statement: I currently or have previously considered the price of the prescription before collecting it							
Total Sample	368	152 (41.31)	59 (16.03)	157 (42.66)	1.29	0.47, 3.55	0.60
LTHC	260	110 (42.31)	42 (41.54)	108 (16.15)			
No LTHC	108	42 (38.89)	17 (15.74)	49 (45.37)			
To what extent do you agree with the below statement: I currently or have previously had to make financial sacrifices in order to afford my prescriptions							
Total Sample	368	88 (23.91)	46 (12.50)	234 (63.59)	2.18	.72, 6.65	.20
LTHC	260	66 (25.38)	30 (11.54)	164 (63.08)			
No LTHC	108	22 (20.37)	16 (14.82)	70 (64.81)			
To what extent do you agree with the below statement: The costs of my prescriptions currently places or has previously placed additional financial burden on me and/or my family							
Total Sample	366	76 (18.31)	67 (20.76)	223 (60.93)	6.87	2.10, 22.5	.001
LTHC	257	65 (25.29)	42 (16.34)	150 (58.37)			
No LTHC	109	11 (10.09)	25 (22.94)	73 (66.97)			
To what extent do you agree with the below statement: The costs associated with my medications currently places or has previously placed emotional stress on myself and/or my family							
Total Sample	367	58 (15.80)	68 (18.53)	241 (65.67)	5.87	1.44, 24.0	0.01
LTHC	258	52 (20.16)	44 (17.05)	162 (62.79)			
No LTHC	109	6 (5.51)	24 (22.02)	79 (72.47)			
Follow-up Q: When I chose not to collect my prescription my health deteriorated Primary question (Have you previously (even just once) elected not to collect a prescription due to the costs associated with it?)†							
Total Sample	86	42 (48.84)	21 (24.42)	23 (26.74)	4.03	0.26, 62.3	0.30
LTHC	59	34 (57.63)	15 (25.42)	10 (16.95)			
No LTHC	27	8 (29.63)	6 (22.22)	13 (48.15)			
Follow-up Q: When I chose not to collect my prescription my health deteriorated Primary question (Have you ever (even just once) altered or deviated from your medication regime due to cost that is, halving medication dose etc?)†							
Total Sample	60	37 (61.67)	13 (21.67)	10 (16.66)	2.30	0.53, 9.95	0.30
LTHC	48	31 (64.58)	9 (18.75)	8 (16.67)			
No LTHC	12	6 (50.00)	4 (33.33)	2 (16.67)			

Note. Relative Risk Ratio (RRR) from multinomial logistic regression comparing level of agreement/awareness between people with LTHCs and people without LTHCs.

* The % level of agreement/disagree is calculated for that specific sample. For example, the level of agreement with the current per-prescribed item price for people with a LTHC is calculated as 63/264.

† Denotes a follow up question for these questions the main question is detailed in parentheses below the question analysed.

### Impact of prescription charges on behaviours

Across both people with and without LTHCs, 41.31% agreed that they had previously considered the price of a prescription prior to collecting it, with no significant difference in the level of agreement between groups (RRR=1.29; 95% CI 0.47, 3.55, p=0.60; see table 2).

23.91% of all participants agreed they had previously had to make financial sacrifices to afford their prescriptions, with no significant difference in the level of agreement between participants with and without an LTHC (RRR=2.18; 95% CI 0.72, 6.65, p=0.20). However, people with an LTHC (vs without) were significantly more likely to agree that prescription charges place additional financial burden (RRR=6.87; 95% CI 2.10, 22.5, p=0.001) on themselves and/or their families (see table 2 for further breakdown).

Regarding emotional burden, 15.80% of all participants agreed that per-prescribed item charges placed emotional burden on themselves and/or their families, with no significant difference in the level of agreement between people with and without LTHCs (RRR=5.87; 95% CI 1.44, 24.0, p=0.01; see table 2).

Most participants (76.29%) indicated that they had never failed to collect a prescription due to the costs associated with it, with no significant difference in the occurrence of non-collection between those with and without an LTHC (OR=1.56; 95% CI 0.75, 3.37, p=0.20; see table 3). A substantial portion (48.84%) of participants who indicated that they had previously not collected a prescription due to cost indicated that they believed their health deteriorated due to not collecting their prescription, with no significant difference in the level of agreement between people with and without LTHCs (RRR=4.03; 95% CI 0.26, 62.3, p=0.30; see table 2).

**Table 3:** Binomial logistic regression analyses examining differences in the impact of prescription charges and the level of awareness of current initiatives to reduce the financial burden of per-prescription charges between participants with and without LTHCs

	N	Yes (n (%))*	No (n (%))*	Binomial logistic regression results (LTHC vs no LTHC)		
				OR	95% CI	P value
Awareness and use of current initiatives to reduce financial burden (PPC)						
Have you ever heard of an NHS Prescription Prepayment Certificate (PPC). A PPC is a certificate that covers all your NHS prescriptions for a set price, like a prescription charge season ticket. Further information regarding the PPC can be found at {Link}.						
Total sample	374	242 (64.71)	132 (35.29)	4.92	2.62, 9.47	<0.001
LTHC	264	197 (74.62)	67 (25.38)			
No LTHC	110	45 (40.91)	65 (59.09)			
Do you have an NHS Prescription Prepayment Certificate (PPC)? AND If/when you were required to pay for your prescriptions did you have an NHS Prescription Prepayment Certificate (PPC)? (for people who indicate they no longer pay)						
Total sample	369	98 (26.56)	271 (73.44)	11.9	4.30, 43.1	<0.001
LTHC	261	90 (34.48)	171 (65.52)			
No LTHC	108	8 (7.41)	100 (92.59)			
Impact of prescription charges on behaviours						
Have you previously (even just once) elected not to collect a prescription due to the costs associated with it?						
Total sample	367	87 (23.71)	280 (76.29)	1.56	.75, 3.37	0.20
LTHC	258	60 (23.26)	198 (76.74)			
No LTHC	109	27 (24.77)	82 (75.23)			
Have you ever (even just once) altered or deviated from your medication regime due to cost that is, halving medication dose etc?						
Total sample	369	62 (16.80)	307 (83.20)	4.36	1.69, 12.8	0.004
LTHC	259	50 (19.31)	209 (80.69)			
No LTHC	110	12 (10.91)	98 (89.09)			

Note.: Odds ratio (OR)OR from binomial logistic regression comparing level of agreement/awareness between people with LTHCs and people without LTHCs.

*The % level of agreement/disagree is calculated for that specific sample. For example, the occurrence of non-collection for people with an LTHC is calculated as 60/258.

LTHCslong-term health conditions

Participants with LTHC (vs without) were more likely to deviate from their medication regime (OR=4.36; 95% CI 1.69, 12.8, p=0.004; see table 3). The majority of participants (61.67%), who disclosed deviating from their medication regime, agreed that their health deteriorated due to lack of medication-taking compliance, with no difference in the level of agreement between people with and without LTHCs (RRR=2.30; 95% CI 0.53, 9.95, p=0.3; see table 2).

### Qualitative findings

Thematic analysis resulted in four overarching themes with various subthemes: (1) the need for re-evaluation; (2) the burden of prescription charges; (3) inconsistencies and inequalities in current policy; and (4) the positive value of prescription charge policy (see table 4 for breakdown of themes and subthemes quotes associated with each theme).

**Table 4:** Thematic analysis results

Theme	Subtheme	Quotes
The need for re-evaluation		
	Reduction in current per-prescribed item charge.	*“The current prescription charge is too high, considering a tax on income already goes into the health system, it is seems ridiculous that a simple prescription can cost this much for someone.”* (30, Participant without a LTHC)*“I think all prescriptions should be free for everyone”*. (62, Participant with a LTHC)*“A small charge is reasonable to prevent misuse but most of the cost should be paid through National Insurance contributions (tax payments towards the NHS).”* (65, Participant without a LTHC)
	Current MedEx criteria are outdated.	*“The policies on prescription charges seemed to be based on rather dated criteria. Patterns of illness, treatments and income policy have all changed radically over the years since the whole development of the NHS.” (Female, 77, Participant with a LTHC*)*“The problem currently is that only the illnesses on the 1960’s medical exemption list get all their medications for free. How can this be fair.”* (Age not disclosed, Participant with a LTHC)*“People with a lifelong health condition which isn't going to improve, they should be entitled to free prescriptions”* (50, Participant with a LTHC)*“All people with chronic illness should be entitled to free prescriptions”* (40, Participant without a LTHC)
	Changes in age base exemption criteria.	*“I believe that it should be means tested, although over 60 I am in a position financially to pay and relieve the burden on the NHS.”* (60, Participant with a LTHC)*“Many people are still working at 60 when prescriptions become free, some in well paid jobs. This seems unfair, my husband was earning over £45K and getting free prescriptions yet another family member earned £25K and had to pay for all his.”* (60, Participant with a LTHC)
The burden of prescription charges		
	Financial burden.	**“***There have been times where I’ve needed 6 months worth of medicines and this is costly and takes up a large percentage of my income.”* (33, Participant without a LTHC)*“Prescriptions are extortionate and the prices are only going to get worse making them unfeasible. I have previously not collected treatments due to the cost, like antibiotics. People not taking tablets as they can't afford them will only add financial burden on the NHS and result in poorer health and even death.”* (37, Participant with a LTHC)
	Emotional burden.	*“I think it’s bad enough to have a condition without having to pay for the privilege of having it”* (61, Participant with a LTHC)*“Enough time and energy is required to deal with combatting and living with the condition itself let alone also having to think about financing prescriptions”* (66, Participant with a LTHC)
Theme	Subtheme	Quotes
Inconsistencies and inequalities in current policy		
	Inconsistencies in policy across the home nations.	*“I find it unfair that Scotland enables their people to all receive free prescriptions, but in England we can't. We should follow in their footsteps.”* (23, Participant with a LTHC)
	Inequalities in the long-term conditions included in the current MedEx criteria.	*“What l don't understand/agree with is the inequity in terms of certain medications for certain chronic conditions being free of charge and others (such as Parkinson’s) not being free of charge. For example, l have a colleague who is diabetic and does not pay for prescriptions. I pay my NI contributions but still have to pay for my medication, which will be the case until I retire. It just feels a bit like adding insult to injury.”* (48, Participant with a LTHC)*“I don’t think the entitlement to free prescriptions is very consistent, across different medical conditions”* (37, Participant with a LTHC)“*The nature of dependency on drugs in certain long-term health conditions needs to be better understood. Yes, it is true that we will not die without them, but we very quickly reach a point where we absolutely cannot function without the medications, in the longer term, not taking my medications (for Parkinson’s) will accelerate physical decline and shorten my lifespan.”*. (55, Participant with a LTHC)
	Inconsistencies in dispensing practices.	*“I have observed variability between Drs for the number of tablets prescribed at one time. One Dr prescribed me a lot, another hardly any but I have to pay the same fee for both amounts. I find this very irritating.”* (53, Participant without a LTHC)
Positive reflections of prescription charge policy		*“Having lived in America, where prescriptions can cost over $1000 every month, the costs in England are wonderful.”* (54, Participant without a LTHC)*“For health reasons, where I wasn’t eligible for NHS treatment, I am aware of the costs of medicines without NHS support. I was fortunate that I could still afford the cost, but knowing the cost of treatment I have had that wasn’t available to me on the NHS I am very grateful for the treatments that are subsidised for me on the NHS.”* (38, Participant with a LTHC)

LTHClong term health conditionNHSNational Health ServiceNINational Insurance

#### The need for re-evaluation

The most commonly discussed theme was the need for the current prescription charge policies to be re-evaluated. While many participants called for a reduction in per-prescritpion charges and the re-evaluation of the current MedEx criteria, other participants proposed changes to age-based exemption criteria (ie, people aged 60+ paying for prescriptions).

##### Reduction in current per-prescribed item charge

Many participants expressed opinions that the current per-prescribed item charge is too high and should be reduced. While some participants simply stated that the current per-prescribed item charge should be reduced and or/entirely removed, others discussed the need for a small fee to reduce misuse, but ultimately agreed the overall fee should be reduced.

##### Current MedEx criteria are outdated

Both people with and without an LTHC discussed that current MedEx criteria are perhaps outdated, and that inequalities in the current criteria could be addressed by revising the criteria. The most common suggestion in relation to revising the current MedEx criteria is that all LTHCs requiring chronic pharmacological treatment should be added to the criteria.

##### Changes in age base exemption criteria

Several participants, who themselves are aged 60+, suggested that people aged 60+ should perhaps be required to pay for their prescriptions should their financial situation allow them to.

### The burden of prescription charges

We found the burden of prescription charges to be twofold: financial and emotional.

#### Financial burden

There was a consensus that per-prescribed item charges heighten feelings of financial burden both to the individual for who the prescription is for but also potentially to the NHS. Participants noted that monthly prescription costs take up a large percentage of their income, and that people not collecting their prescriptions due to cost will likely result in poorer health outcomes leading to an increased need for additional health services.

#### Emotional burden

Although quantitatively fewer, compared with expressions of the occurrence of financial burden, some participants expressed feelings of distress due to prescription charges. Participants noted that navigating living with an LTHC can be emotionally distressing, and that additional pressures of having to finance prescriptions may make an already distressing circumstance much worse.

#### Inconsistencies and inequalities in current policy

Many participants reflected on the lack of consistency and occurrence of inequalities in current per-prescribed item charge policy and the application of such policies. In particular, inconsistencies in polices applied across home nations, inequalities in the long-term conditions included in MedEx criteria and inconsistencies in dispensing practices were discussed.

#### Inconsistencies in policy across the home nations

Participants expressed feelings of dissatisfaction and confusion as to why the policies in place in England are not comparable to those in place in other home nations (Scotland and Wales; where prescriptions are free of charge for everyone).

#### Inequalities in the long-term conditions included in the current MedEx criteria

Participants highlighted inequalities in the current MedEx criteria. Participants expressed confusion as to why some LTHCs are included in such criteria while others are not. Some participants questioned whether such inequalities may perhaps stem for a lack of understanding regarding some LTHCs.

### Inconsistencies in dispensing practices

Dispensing practices were oftentimes variable within localised healthcare settings. This variability in practice may ultimately lead to variability in the prescription charges people face.

### Positive reflections of prescription charge policy

Several participants expressed positive sentiments towards prescription charges and not having to pay full medication costs. Some participants reflected on the circumstances in other countries such as the USA and found the circumstances in England to be positive in comparison.

## Discussion

This study is the first to examine the acceptability of current UK prescription charge policy, the impact of prescription charges and the level of awareness of current initiatives to reduce the financial burden of per-prescribed item charges in both people living with and without LTHCs.

Over half (53.21%) of all participants disagreed with current per-prescribed item charges, with no differences in the level of agreement being observed between people with (vs without) LTHCs. Importantly, the lack of difference between people with and without LTHCs opinions may indicate that lack of support for current per-prescribed item charge policy is not unique to people with LTHCs. When asked to reflect on the fee they would find reasonable, some participants stated that prescriptions should be free, while others acknowledged the need for a small fee to reduce misuse. It is, however, important to note that a small proportion of participants (25.67%) agreed that the current cost is fair and reasonable. Furthermore, several participants indicated that they find prescription charge policy in England to be favourable compared with other developed countries, namely the USA.

41.31% of participants agreed that they had previously considered the price prior to collection and 23.91% of participants agreed that they had had to make financial sacrifices to afford their prescriptions, with no significant differences in the level of agreement being observed between people with (vs without) LTHCs. In this study sample, there were no significant differences between participant groups (LTHCs vs no LTHC) in terms of annual household income. Therefore, it is unlikely that the lack of difference between people with and without LTHCs can be accounted for by any one group having significantly greater ability to finance such charges. Thus, our results may suggest that per-prescribed item prescription charge policy may impact both participants with an LTHC and those without an LTHC to a similar extent.

Prior research, although primarily non-UK based, has shown that medication costs may be significant barriers to medication adherence.^4 15^ Congruent with these observations, 23.71% of participants reported not collecting a prescritpion and 16.80% of participants reported having deviated from their prescribed medication regime (eg, skipping doses or half-dosing) due to the associated per-prescribed item costs. Medication non-adherence can lead to reductions in functional abilities and quality of life, the occurrence of additional health complication, premature death and increased use of medical resources (eg, hospitalisation).^16^,^18^ Thus, per-prescribed item cost may to some extent influence the likelihood of an individual experiencing poorer health outcomes. It is, however, important to note that a wide range of factors, beyond per-prescribed item charges, influence medication adherence.^19^ Thus, these assumptions should be treated with a degree of caution. Furthermore, while per-prescribed item costs aim to produce income for the NHS, non-adherence behaviours may ultimately result in greater costs to the NHS. Indeed, it has been estimated that medication non-adherence in asthma, type 2 diabetes, high cholesterol/coronary heart disease, hypertension and schizophrenia may cost the NHS up to £930 million per annum,^20^ and ~4.60% of total global health expenditures could be avoided with better medication adherence.^21^

It is, however, important to reflect on the differences observed between people with and without LTHCs with respect to prescription collection and medication regime adherence. Specifically, although people with LTHCs were more likely to deviate from their medication regime, we did not observe a significant difference in the occurrence of prescription non-collection between people with and without LTHCs. Medication regimes for people living with LTHCs tend to be more complex and lengthy. Thus, it may be that the non-adherence with medication regimes reported here was in part due to the complexity of the medication regime as opposed to the cost associated with the prescription. Indeed, prior evidence has shown that medication regime complexity negatively impacts the level of medication adherence in people with multiple conditions.^22^ However, the question posed to participants in the present study was ‘*Have you ever (even just once) altered or deviated from your medication regime due to cost*’. As the question posed specifically asked participants to reflect on the impact of cost, this assumption may be unlikely.

Prior evidence suggests that emotional burden and poorer mental health outcomes may arise due to medical costs when individuals have to forgo other necessities, such as food, medical care, housing and basic utilities because of insufficient financial.^23 24^ In line with this evidence, a small proportion of participants (15.80%) reported that per-prescribed item charges placed emotional burden on themselves, with both people with and without an LTHC being equally likely to agree to experiencing emotional burden. In this study, 63.59% of participants indicated that they had not had to make financial sacrifices to afford their prescriptions. Thus, it is perhaps unsurprising that, in this sample, the proportion of participants reporting the occurrence of emotional burden is low. Future research would benefit from recruiting a sample of participants who are required to make financial sacrifices to afford their prescription to fully ascertain the emotional impact of per-prescribed item charges in the UK.

Prior evidence has shown that people with LTHCs in the UK find it unfair that they have to pay for their prescriptions.^4^ Building on this evidence, we showed that people with LTHCs found the current MedEx criteria to be outdated and inequitable, with many participants calling for a review of these criteria to cover a larger range of LTHCs.

Just over one-third of all participants had never heard of the PPC, with people living with LTHCs, who are more likely to benefit from this initiative, being more likely to be aware of the PPC. However, 25.38% of people with an LTHC were still unaware of the PPC. Awareness of the PPC, within the sample recruited here, appeared to be driven by word of mouth with just under a third of respondents (31.00%) citing the source of awareness being friends/family. While word of mouth is recognised as an effective means of ‘advertising’,^25^ prior evidence suggests that healthcare provision awareness is influenced by socioeconomic factors, including educational attainment.^26 27^ Thus, reliance on this means of advertising may give rise to inequitable access to this healthcare provision. Therefore, it would perhaps be beneficial to move away from a reliance on word-of-mouth advertising and increase visual advertising presence in locations that individuals with LTHCs will be likely to attend (ie, general practitioner surgery and hospital waiting rooms and pharmacies).

These findings have important implications for prescription charge policy. Over half of the sample expressed a lack of support with the current prescription charges. Moreover, as medication non-adherence may influence the health outcomes for the individual for which the prescription relates to, and increased financial burden on the NHS,^18 19^ reducing the financial barrier of prescription charges will also be important to accommodate all needs. Due to these factors, it would be beneficial for current prescription charge policy to be reviewed and potentially reduced. While the reduction of per-prescribed item charges would still leave England as the only UK nation currently paying for prescriptions, the abolishment of prescription charges would align policy in England with other home nations. Within this study, people with LTHCs responses differed from those without LTHCs only with regard to the occurrence of financial burden and medication adherence. Thus, it may be that any changes to per-prescribed item charges will impact the wider population not just people with LTHCs. There is a lack of consistency in the clinical conditions that are eligible for medical exemption and those that are not. Re-evaluating the MedEx criteria and justifying the clinical conditions included would provide clarity and reduce systematic inequalities. We observed that public awareness of initiatives to reduce prescription charges is low to moderate and hence efforts should be made to increase public awareness of this.

While this is an England-centric study, our findings may have important implications for other countries that are currently considering alterations to prescription charge policy. For example, we showed that people with LTHCs find the current MedEx criteria to be outdated and inequitable and questioned whether such inequalities may perhaps stem for a lack of understanding regarding some LTHCs. Thus, our findings suggest that countries who are currently developing an exemption criterion (eg, The national pharmacare policy of Canada^28^) should ensure that any exemption criterion is thoroughly planned relating to medication reliance in clinical conditions and that people living with LTHCs should be consulted during such development processes.

This study has several strengths with the inclusion of free-text qualitative responses, responses across the UK, and the representation of a wide array of LTHCs. However, this study is not without limitations. First, caution must be applied when generalising the results of this study. This study recruited a volunteer sample. Although the sample is diverse in terms of geographical spread of participants, IMD values and annual household income, there is a greater proportion of females, people from white ethnic groups and highly educated people. Healthcare provision awareness^26 27^ and healthcare use^29^ appear to be influenced by socioeconomic and demographic factors, including educational attainment, ethnicity, income and IMD. Therefore, further studies recruiting more ethnically, and educationally diverse samples are required to better ascertain the perceptions and impact of prescription charge policy across the UK public. Second, as this study recruited a volunteer sample, the issue of self-selection bias must not be overlooked. It is not unreasonable to assume that several participants were elected to partake in this survey due to an individual preoccupation with medication charges. Thus, the findings obtained here may not be representative of people to whom prescription medications are less of a concern. Third, this study employed self-report methods. It is well established that participants oftentimes inaccurately report their answers to provide the most socially agreeable responses.^30^ While this study was completed anonymously, thereby reducing the chance of social desirability bias, it may be that some participants will have modified their answers in order to conform to what they believe to be socially agreeable (eg, under-reporting the true financial impact of per-prescribed item charges). Therefore, further studies that analyse individual behaviour data, such as pharmacy collection data, would be useful. Finally, this study did not directly assess the impact of polypharmacy (ie, the number of prescribed medications) and multimorbidity (the number of LTHCs), and therefore conclusions cannot be drawn on the impact of the level of reliance of prescription charges on individuals’ perceptions. Further studies that directly investigate the impact of polypharmacy and multimorbidity are required.

## Conclusion

To conclude, the present study showed that both people with and without LTHCs expressed a lack of support with current per-prescribed item charges and the impact of these charges is comparable between both groups. Although initiatives are in place to reduce the financial burden of per-prescribed item charges, public awareness and use of this initiative are moderate to low. Moreover, there is a consensus of perceived inequalities both in the clinical conditions covered by the MedEX criteria and between the policies employed in England compared with other home nations. Based on these findings, we recommend that the current per-prescribed item charge and the clinical conditions covered under MedEX are reconsidered, and advertising of the PPC is increased.

## supplementary material

10.1136/bmjopen-2024-085345 online supplemental file 1

## Data Availability

Data are available in a public, open access repository.
