# Supplementary material for: Prescription charge policy acceptance among UK adults with and without long-term health conditions: a mixed-method survey
Source: BMJ Open. 2024 Sep 24;14(9):e085345. doi: 10.1136/bmjopen-2024-085345 (PMC11423742; doi:10.1136/bmjopen-2024-085345)
Supplement: online supplemental file 1 [file bmjopen-14-9-s001.pdf]

Supplementary Materials

Contents

Study Protocol (Including Table S1- S5) 2
Exploratory Analyses (Including Tables S6 – S8) 16
Table S9: Breakdown of long-term health conditions reported. 20

<u>Study Protocol</u>

Contents

1. Background
2. Methods
   2.1 Participants
      2.1.1 Sample Size Calculation
      2.1.2 Inclusion/ Exclusion Criteria
      2.1.3 Recruitment Strategy
   2.2 Survey Measures
      2.2.1 Demographic Variables
      2.2.2 Health background variables
      2.2.3 Prescription medication taking behaviour variables
      2.2.4 Acceptability and perceptions of prescription charge policy variables
      2.2.5 Awareness and use of current initiatives to reduce financial burden (PPC) variables
3. Procedure
   3.1 Ethics
   3.2 Procedure
4. Data Analysis
   4.1 Data Transformation
      4.1.1 IMD Values
      4.1.2 Participant group level categorisation
      4.1.3 PPC initiative use
      4.1.4 Likert responses
      4.1.5 Three level ("Yes", "No", "Prefer not to say") responses
   4.2 Statistical Analyses
      4.2.1 Missing Data
      4.2.2 Primary Quantitative Analysis
      4.2.3 Exploratory Quantitative Analysis
      4.2.4 Qualitative Analysis
5. References

1. Background

Since their introduction in 1952, per-prescription charges in England have continually risen, with the latest charge of £9.65 per prescription being brought into effect in April 2023 [1]. A system of exemptions enables people in defined categories (people under the age of 16, people aged 16-18 in full time education, and people over the age of 60, people who are pregnant or have had a baby in the last 12 months (MatEx), people who receive certain state benefits, and people living with *specific* medical conditions (MedEx)) to receive prescription medications for free. However, a substantial number of long-term health conditions, that require chronic pharmacological intervention, are omitted from such criteria. Resultantly, many people living with various long-term health conditions that require chronic pharmacological intervention are required to pay for their prescriptions.

To mitigate the financial burden of per-prescription charges, particularly for people living with long-term health conditions, the Department of Health and Social Care introduced a Prescription Prepayment Certificate (PPC). PPC's enable individuals to make a one-off payment that covers all prescription charges for the following year/ 3-month period, thus limiting the amount people pay annually for prescriptions. Theoretically this initiative will substantially reduce financial burden for people living with long-term health conditions. However, the effectiveness of this initiative is contingent upon people living with long-term health conditions 1) being aware of the initiative and 2) using the initiative.

Presently, understanding of the perception of current prescription charge policy and the impact of per-prescription changes, particularly on people with long-term conditions, are limited. Therefore, the present cross-sectional mixed-methods survey aims to (1) further our understanding of the acceptability of prescription charge policy and the impact of prescription charges on both people living with and without long-term health conditions in the UK, and (2) ascertain the level of awareness of current initiatives to reduce the financial burden of per-prescription charges. To do so, both people living with and without long-term health conditions will be asked to share their opinions and experiences regarding the current per-prescription fee, the impact of prescription charges and engagement with the PPC.

2. Methods

2.1. Participants

2.1.1 Sample Size Calculation

G*Power software [2] was used to perform an a priori power analysis to ascertain the required sample size to achieve adequate power. The smallest acceptable power ($1 - \beta$) was set at .80 and the significance level (α) was set to .05. There is no previously published evidence available to inform the anticipated proportion of people without long-term health conditions who disagreed with the policy. Therefore, in accordance with the National Institute of Health Research, research and design guidance [3], to increase sample size H_0 was set at an arbitrary value of 50%. To detect statistically small differences in the proportion (15%) of agreement or disagreement between those with vs. without a long-term health condition, H_1 was set at 65%. For the frequentist parameters defined, a total sample size of N =380, with an approximate ~50% of participants self-identifying as having long-term health conditions and ~50% self-identifying as not having long-term health conditions, is required.

2.1.2 Inclusion Criteria

Participants will be categorised into two conditions: 1) people living with long term health conditions and 2) people who do not have a long-term health condition. An equal number of participants (n = 190) will be recruited to each condition.

People living with long term health conditions
Inclusion criteria
- Self-identify living with a long-term health condition (any condition)
- Aged 18+
- Living in the UK (England, Scotland, Wales, Northern Ireland)
- Fluent in English (as identified as the ability to read and respond to the questions)
- Have the capability to complete an online survey (access to IT and sufficient capacity to effectively complete the survey).

People without long term health conditions
Inclusion criteria
- Do not identify as living with a long-term health condition
- Aged 18+
- Living in the UK (England, Scotland, Wales, Northern Ireland)
- Fluent in English (as identified as the ability to read and respond to the questions)
- Have the capability to complete an online survey (access to IT and sufficient capacity to effectively complete the survey).

2.1.3 Recruitment Strategy

Participants will be recruited via advertisements on social media (e.g. X (formerly twitter) and Facebook), and emails to the research teams local/national research participation networks (i.e. MR's Parkinson's research interest data). Parkinson's UK will also aid in recruitment by posting survey adverts on official their Twitter, LinkedIn and Facebook pages. We aim to have all participants recruited by December 1st, however, if the required sample has not been recruited by this date the survey will remain open with the latest survey closing date being 31.12.2023.

2.2.Survey Variables

2.2.1 Demographic Variables

Data relating to participants demographic and socioeconomic characteristics will be obtained. To obtain such data 5 multiple choice and five free text response questions relating to age, gender, ethnicity, employment status, occupation, educational attainment, household income, household occupancy and location of residence, will be included (See Table 1 for full questions).

Table S1. Demographic variables questions.

Question	Response options
1) How old are you in years?	Free Text
2) What gender do you identify as?	Male<br>Female<br>Non-binary/ Third Gender<br>Prefer not to say
3) Please specify your ethnicity	White- English/Welsh/Scottish/Northern Irish<br>White- Irish<br>White- Any other white background<br>Mixed- White and Black Caribbean<br>Mixed- White and Black African<br>Mixed- White and Asian<br>Mixed- Any other mixed/multiple ethnic background<br>Asian- Indian<br>Asian- Pakistani<br>Asian- Bangladeshi<br>Asian- Chinese<br>Asian- Any other Asian background<br>Black- Black British<br>Black- Black African<br>Black- Black Caribbean<br>Black- Any other Black background<br>Any other Ethnic group<br>Prefer not to say
4) Considering your current employment status are you:	Working full time<br>Working part time<br>Retired<br>Out of Work<br>Student<br>Prefer not to say
5) What is your current occupation?	Free text
6) What is your highest level of educational attainment?	GCSE or equivalent<br>AS/ A levels or equivalent<br>Degree level or above (e.g. degree, foundation degree, HND or HNC level 4 or above)<br>No qualifications<br>Prefer not to say

	Question	Response options
7)	What is your total annual household income before tax and deductions?	£0 - £10,000<br>£10,001- £20,000<br>£ 20,001 - £30,000<br>£ 30,001 - £40,000<br>£ 40,001 - £50,000<br>£ 50,001 - £60,000<br>£ 60,001 - £70,000<br>£ 70,001 - £80,000<br>£ 80,001 - £90,000<br>£ 90,001 - £100,000<br>£ 10,001 +<br>Prefer not to say
8)	How many people aged 14 years and over do you currently live with in the same household, including yourself?	Free text
9)	How many children aged less than 14 years do you currently live with in the same household?	Free text
10)	Please detail the first 3 digits of your permanent post code.<br>(note. this will be used to locate your overall geographical region, however, we cannot locate a specific street or house using this information).	Free text

2.2.2 Health Background variables

Participants will be asked to self-identify whether they are living with any long-term health conditions. To do so participants will be asked to confirm ("yes", "no", "prefer not to say" whether they are living one of eleven pre-set long-term health conditions or another non-specified long-term health conditions. Should participants indicate they are living with a long term health condition they will be prompted to disclose the nature of this condition (See Table 2 for full questions).

Table S2. Background health variables questions.

Question	Response options
11) Do you have a current diagnosis, or a history of a diagnosis, of the following health conditions<br><br>a. *Neurological conditions (e.g., Parkinson's disease, Parkinsonism disorders)*<br>b. *Cognitive impairment (e.g., Mild cognitive impairment, dementia)*<br>c. *Psychiatric conditions (e.g., depression, anxiety, and bipolar)*<br>d. *Rheumatic illnesses (e.g., Arthritis),*<br>e. *Autoimmune disorders (e.g., lupus)*<br>f. *Diabetes*<br>g. *Chronic obstructive pulmonary disease*<br>h. *Hypertension*<br>i. *Epilepsy*<br>j. *Blood disorders*<br>k. *Cystic fibrosis*<br>l. *Other condition not specified.*	Yes,<br>No,<br>Prefer not to say
12) If you answered yes to any of the above health conditions, or have an alternative health condition not specified, please provide details of the specific condition.	Free text

2.2.3. Prescription medication taking behaviours

Two binary response questions, with follow up free-text questions, will be included to ascertain participants prescription medication taking behaviours (See Table 3 for full questions).

Table S3. Prescription medication taking behaviour questions.

Question	Response options
13) Are you currently prescribed, and taking, any long-term medications?	Yes,<br>No,<br>Prefer not to say
a) If yes to Q1: On average how many medications are you prescribed?	Free text
b) If yes to Q1: On average how many tablets would you take a day?	Free text
14) Do you currently have to pay for your prescriptions?	Yes,<br>No,<br>Prefer not to say
a) If yes to Q2: In a typical month how much would you spend on medications?	Free text
b) *If no to Q2:* Please select the reason for which you do not have to pay for your prescriptions.	I am over 60,<br>I am pregnant or have had a baby in the last 12 months,<br>I have a specified medical condition and have a valid medical exemption certificate (MedEx),<br>I have a continuing physical disability that prevents you going out without the help from another person, and have a valid medical exemption certificate (MedEX),<br>I hold a valid war pension exemption certificate and the prescription is for your accepted disability<br>I receive: income support, income-based jobseeker's allowance, income-related employment and support allowance, pension credit guarantee card or universal credit,<br>I have a valid NHS tax credit exemption certificate,<br>I have a valid NHS certificate for full help with health costs (HC2),<br>Prefer not to say
c) If no to Q2: When you used to pay for your medication/ if you have ever paid, what was the maximum amount of money per month you would spend on medications?	Free text

2.2.4 Awareness and use of current initiatives to reduce financial burden (PPC) variables.

Participants awareness and use of the PPC initiative will be assess through three multiple response questions (See Table S4 Qs1, 2, and 3). These core questions will be followed up with free text response questions to allow participants to detail how they became aware of the PPC and why they chose to engage with the PPC initiative (See Table S4 for full questions).

Table S4. Awareness and current use of the PPC initiative questions.

	Question	Response options
1)	Have you ever heard of an NHS Prescription Prepayment Certificate (PPC)?	Yes,<br>No,<br>Prefer not to say
	a) If yes to Q3: How did you find out about the Prescription Prepayment Certificate (PPC)?	Free text
2)	Do you currently have an NHS Prescription Prepayment Certificate (PPC)?	Yes,<br>No,<br>Prefer not to say
	a) If yes to Q4: Have you always had an NHS Prescription Prepayment Certificate (PPC)	Yes,<br>No,<br>Prefer not to say
	b) If no to Q4: Please can you provide a little more information as to when you chose to get a Prescription Prepayment Certificate (PPC) and why.	Free text
	c) If no to Q4: Is there any reason why you do not have an NHS Prescription Prepayment Certificate (PPC)?	Free text
3)	*If no to (do you currently pay for your prescriptions)* If/When you were required to pay for your prescriptions did you have an NHS Prescription Prepayment Certificate (PPC)?	Yes,<br>No,<br>Prefer not to say
	a) If yes to Q5: If/when you paid for your prescriptions did you always have an NHS Prescription Prepayment Certificate (PPC)	Yes,<br>No,<br>Prefer not to say
	b) *If yes to Q5:* Please can you provide a little more information as to when you chose to get a Prescription Prepayment Certificate (PPC) and why.	Free text
	c) If no to Q5: Is there any reason why you do not have an NHS Prescription Prepayment Certificate (PPC)?	Free text

2.2.5 Acceptability and perceptions of prescription charge policy variables

Participants acceptability and perceptions of current prescription charge policy will be assessed using five, five-point Likert scale questions with rating scales from strongly disagree to strongly agree. Two additional binary response questions, with follow up five-point Likert scale questions, will also be included to assess the impact of per-prescription charges on adherence to prescribed medication regimes. Furthermore, one free text response questions will be included to allow participants to qualitatively express their opinions on these policies further (See Table S5 for full questions).

Table S5. Perceptions of prescription charge policy questions.

Question	Response options
1) The current price of prescriptions is fair and reasonable	Strongly Agree<br>Agree<br>Neither agree nor disagree<br>Disagree<br>Strongly Disagree<br>Prefer not to say
a) *If disagree/strongly disagree selected to Q1:* Please can you tell us what price you think would be reasonable.	Free text
2) I currently or have previously considered the price of the prescription before collecting it	Strongly Agree<br>Agree<br>Neither agree nor disagree<br>Disagree<br>Strongly Disagree<br>Prefer not to say
3) I currently/ previously have had to make financial sacrifices to afford my prescriptions	Strongly Agree<br>Agree<br>Neither agree nor disagree<br>Disagree<br>Strongly Disagree<br>Prefer not to say
4) The costs of my prescriptions currently places or has previously placed additional financial burden on me and/or my family	Strongly Agree<br>Agree<br>Neither agree nor disagree<br>Disagree<br>Strongly Disagree<br>Prefer not to say
5) The costs associated with my medications currently places or has previously placed emotional stress on myself and/or my family	Strongly Agree<br>Agree<br>Neither agree nor disagree<br>Disagree<br>Strongly Disagree<br>Prefer not to say
6) Have you previously (even just once) elected not to collect a prescription due to the costs associated with it?	Yes<br>No<br>Prefer not to say

Question	Response options
a) *If yes to Q6* When I chose not to collect my prescription my health deteriorated	Strongly Agree<br>Agree<br>Neither agree nor disagree<br>Disagree<br>Strongly Disagree<br>Prefer not to say
7) Have you ever (even just once) altered or deviated from your medication regime due to cost i.e., halving medication dose etc?	Yes<br>No<br>Prefer not to say
a) *If yes to Q7* When I chose not to collect my prescription my health deteriorated	Strongly Agree<br>Agree<br>Neither agree nor disagree<br>Disagree<br>Strongly Disagree<br>Prefer not to say
8) Do you have any other comments or thoughts about prescription charge policy?	Free text

3. Procedure

3.1. Ethics

This study received favourable ethical approval from both the University of Liverpool [Ref: 12769] and the Division of Health Research of Lancaster University [Ref: 3622].

3.2. Procedure

Completion of this study will occur online only and will take approximately 20-30 minutes to complete. To commence the survey, participants will first be presented with the information sheet, detailing the study requirements and researcher contact details should they wish to contact us for further information. Participants will then be asked to provide written informed consent, through ticking each statement to indicate agreement. Please note, the consent form is restricted such that participants cannot pass on to the main survey questions until agreement is provided for each of the 10 statements individually. On completion of the consent form participants will be instructed to create a unique ID number, which they will be asked to store and quote during any correspondence with the research team. Once the participant has developed a unique ID number they will then be presented with the aforementioned survey questions (see Figure 1 for flowchart of study administration).

Figure 1. Study Flowchart and anticipated task completion times.

1. Information sheet (~ 3 minutes)

↓

2. Informed consent (~2 minutes)
3. Unique ID number development (~2 minutes)
4. Demographic questions (~ 3 minutes)
5. Health background questions (~5 minutes)
6. Prescription medication taking behaviours questions(~ 5 minutes)
7. Acceptability and perceptions of prescription charge policy questions (~5 minutes)
8. Debrief (~ 5 minutes)

4. Data analysis

4.1. Data Transformation

The code used to employ data transformations will be freely shared on completion of the final analysis.

4.1.1 IMD Value

IMD values, for each participant, will obtained by inputting postcode data into the Ministry of Housing, Communities & Local Government English indices of deprivation 2019 Postcode Lookup (https://imd-by-postcode.opendatacommunities.org/imd/2019).

4.1.2 Participant group level categorisation

Participants will be categorised as either living with a long-term health condition or not based on responses to the health background variables (See Table 2). Specifically, participants who

responded ‘yes’ to any one of the pre-set long-term health conditions or disclosed an alternative non-specified long-term health were classified as having a long-term health condition, and those who selected ‘No’ to all pre-set long-term health conditions and did not specify an alternative long-term health condition were classified as not having a long-term health condition. For this analysis, we will not classify participants who disclosed that they were living with multiple long-term health conditions as a distinct group.

4.1.3 PPC initiative use

To capture PPC initiative use both in people who are currently paying for their prescriptions and people who no long pay, use of the PPC initiative will be assed through two distinct questions 1) Do you currently have an NHS Prescription Prepayment Certificate (PPC)? and 2) *If no to (do you currently pay for your prescriptions)* If/When you were required to pay for your prescriptions did you have an NHS Prescription Prepayment Certificate (PPC)?. Participants who respond ‘yes’ to either of the above questions will be classified as having made use of the PPC initiative, and those who selected ‘No’ to either of the above questions will be classified as having not used the PPC initiative.

4.1.4 5- point Likert responses

Participants’ 5-point Likert scale responses will be recoded into three categories: disagree (for “strongly disagree” and “disagree”, neutral (for “neither agree nor disagree”) and agree (for “agree” and “strongly agree”). To maximise the amount of data available for analysis “prefer not to say” responses were omitted for the specific question, to which they answered, “prefer not to say”, analysis only.

4.1.5 Three level (“Yes”, “No”, “Prefer not to say”) responses

Participants three level responses (“Yes”, “No”, “Prefer not to say”) will be recoded into a binary yes no variable. In doing so “prefer not to say” responses will omitted for that specific question.

4.2 Statistical Analyses

4.2.1 Missing Data

To maintain participants right not to provide data for any given question the survey will be formatted to allow submission with items not being answered (i.e. forced choice formatting will not be applied). For all primary analyses the entire sample who have data available for the given outcome variable will be analysed. To maximise the quantity of data available participants with missing data, and participants who responded “prefer not to say”, will be excluded from the analysis for which they have missing data only.

4.2.2 Primary Quantitative Analysis

Multinomial logistic regressions, controlling for age, gender, ethnicity, education, household income and IMD, will be applied examine whether participants with a long-term health condition perceptions of current prescription charge policy (acceptability and perceptions of prescription charge policy questions 1,2,3,4 and 5) significantly differ from participants without long-term health conditions. The likelihood of a given participant selecting disagree or agree, relative to neutral, will be examined. Multinomial logistic regression findings

will presented as relative risk ratio (RRR) along with 95% confidence intervals (CI) and p-value.

Binary logistic regressions, controlling for age, gender, ethnicity, education, household income and IMD, will be applied to investigate the impact of per-prescription charges on prescription medication adherence and the awareness and use of the PPC initiative. Binary logistic regression findings will be presented as odds ratios (OR) along with 95% confidence intervals (CI) and p-value.

To account for multiple comparisons and reduce the likelihood of Type I error, the Bonferroni correction [4,5] will be applied across all logistic regressions (both binary and multinomial). Eleven logistic regressions (7 multinomial logistic regressions and 4 binary regressions) will be conducted, therefore a significance level of $p < 0.0045$ (.05 / 11) will be applied.

4.2.3 Exploratory Quantitative Analysis

In this survey we will recruit both people who are currently paying and people who are not currently paying for their prescriptions. To ascertain whether participants perceptions of current prescription charge policy are influenced by whether they are currently required to pay or not we will conduct additional exploratory analyses including *only* participants who are currently paying for their prescriptions. Specifically, we will conduct all planned primary analyses with *only* participants who are currently paying for their prescriptions. The statistical analyses and planned corrections applied to the exploratory quantitative analyses will be the same as those applied to the primary quantitative analysis.

4.2.4 Qualitative Analysis

Qualitative free text responses will be analysed using inductive thematic analysis informed by Braun and Clarke [6]. All free text responses will be independently coded by two research team members (MR and MP). On completion of the initially coding MR and MP will jointly discuss emerging themes and generate overarching themes and sub-themes. The agreed overarching themes and sub-themes will then be discussed with the wider research team.

5. References

1. Department of Health and Social care. (2023, March 9) *NHS prescription charges from 1 April 2023*. Gov.uk. https://www.gov.uk/government/speeches/nhs-prescription-charges-from-1-april-2023
2. Faul, F., Erdfelder, E., Lang, A. G., & Buchner, A. (2007). G*Power 3: A flexible statistical power analysis program for the social, behavioral, and biomedical sciences. *Behavior Research Methods, 39*(2), 175–191. https://doi.org/10.3758/BF03193146
3. NIHR RDS for the East Midlands, The NIHR RDS for Yorkshire and the Humber [Internet]. 2009 [cited 2023 Dec 10]. Available from: https://www.bdct.nhs.uk/wp-content/uploads/2019/04/Sampling-and-Sample-Size-Calculation.pdf
4. Lee, S., & Lee, D. K. (2018, Oct). What is the proper way to apply the multiple comparison test? Korean J Anesthesiol, 71(5), 353-360. https://doi.org/10.4097/kja.d.18.00242
5. Sedgwick, P. (2012). Multiple significance tests: the Bonferroni correction. BMJ, 344, e509. https://doi.org/10.1136/bmj.e509
6. Braun, V., & Clarke, V. (2006). Using thematic analysis in psychology. Qualitative Research in Psychology, 3(2), 77-101. https://doi.org/10.1191/1478088706qp063oa

Exploratory Analyses

The primary analysis included all participants irrespective of whether they are currently required to pay per-item prescription charges. To explore whether the facet of currently pay prescription charges (vs. not) influenced the results, additional exploratory analyses including only participants who are currently paying for their prescriptions were conducted. Please see Table S7 for full results.

Overall, the findings obtained from analyses considering only participants who currently pay for their prescriptions are largely comparable to findings obtained from analyses considering both participants who currently pay and those who do not currently pay. Specifically, the only difference findings observed was in the full sample analysis, participants with LTHCs *were more likely* to agree that prescription charges place additional financial burden on themselves and/or their family. However, in the people who currently pay only analysis participants with LTHCs *were not more likely* to agree that prescription charges place additional financial burden on themselves and/or their family (See Table S8 for full comparison between analyses).

Table S6. Exploratory multinomial logistic regression analyses (analysis including only participants who currently pay per-item prescription charges)*.

	n	Agree (n(%))	Neutral (n(%))	Disagree (n(%))	*Multinomial logistic regression results (LTHC v No LTHC)* Relative risk ratio (RRR)	95% CI	*p*
**Acceptability of per prescribed item charge policy**							
To what extent do you agree with the below statement: The current price per prescribed item (£9.65) is fair and reasonable							
Total Sample	197	51 (25.89)	35 (17.77)	111 (56.34)			
LTHC	125	31 (24.80)	26 (20.80)	68 (54.40)	.79	.29, 2.18	.70
No LTHC	72	20 (27.78)	9 (12.50)	43 (59.72)			
**Impact of prescription charges on behaviours**							
To what extent do you agree with the below statement: I currently or have previously considered the price of the prescription before collecting it							
Total Sample	195	105 (53.85)	23 (11.79)	67 (34.36)			
LTHC	123	71 (57.72)	14 (11.38)	38 (30.89)	.77	.18, 3.21	.70
No LTHC	72	34 (47.22)	9 (40.28)	29 (12.50)			
To what extent do you agree with the below statement: I currently or have previously had to make financial sacrifices in order to afford my prescriptions							
Total Sample	195	57 (29.23)	18 (9.23)	120 (61.54)			
LTHC	124	42 (33.87)	10 (8.06)	72 (58.06)	1.97	.41, 9.44	.40
No LTHC	71	15 (21.13)	48 (67.60)	8 (11.27)			
To what extent do you agree with the below statement: The costs of my prescriptions currently places or has previously placed additional financial burden on me and/or my family							
Total Sample	196	46 (23.47)	31 (15.82)	119 (60.71)			
LTHC	124	39 (31.45)	15 (12.10)	70 (56.45)	6.49	1.47, 28.7	.01
No LTHC	72	7 (9.72)	16 (22.22)	49 (68.06)			
To what extent do you agree with the below statement: The costs associated with my medications currently places or has previously placed emotional stress on myself and/or my family							

	n	Agree (n(%))	Neutral (n(%))	Disagree (n(%))	*Multinomial logistic regression results (LTHC v No LTHC)* Relative risk ratio (RRR)	95% CI	*p*
Total Sample	196	38 (19.39)	29 (14.79)	129 (65.82)			
LTHC	124	34 (27.42)	15 (12.10)	75 (60.48)	19.10	2.03, 178	.01
No LTHC	72	4 (5.56)	14 (19.44)	54 (54.00)			
***→ *Follow up Q: When I chose not to collect my prescription my health deteriorated*							
Primary question (Have you previously (even just once) elected not to collect a prescription due to the costs associated with it?)							
Total Sample	60	32 (53.33)	12 (20.00)	16 (26.67)			
LTHC	41	26 (63.41)	10 (24.39)	5 (12.20)	1.53	.02, 136	.90
No LTHC	19	6 (31.58)	2 (10.53)	11 (57.89)			
***→ *Follow up Q: When I chose not to collect my prescription my health deteriorated*							
Primary question (Have you ever (even just once) altered or deviated from your medication regime due to cost i.e. halving medication dose etc??)							
Total Sample	41	27 (65.86)	8 (19.51)	6 (14.63)			
LTHC	33	23 (69.69)	6 (18.19)	4 (12.12)	1.92	.28, 13.2	.50
No LTHC	8	4 (50.00)	2 (25.00)	2 (25.00)			

*Note. As these additional analyses were exploratory and will be reflected on only in the sense of ascertaining whether the facet of currently pay prescription charges (vs. not) influenced the results, they were not included in the original Bonferroni p value correction. However, the Bonferroni correction adhered to in the main analyses ($p < 0.005$ (.05 / 9)) was applied when interpreting the results presented here.

Table S7. Binomial logistic regression analyses examining differences in the impact of prescription charges and the level of awareness of current initiatives to reduce the financial burden of per-prescription charges between participants with and without LTHCs. *

	n	Yes (n(%))	No (n(%))	*Binomial logistic regression results (LTHC V No LTHC)* Odds Ratio (OR)	95% CI	*p*
**Impact of prescription charges on behaviours**						
Have you previously (even just once) elected not to collect a prescription due to the costs associated with it?						
Total Sample	193	61 (31.61)	132 (68.39)			
LTHC	122	42 (34.43)	80 (65.57)	1.44	.76, 2.78	.30
No LTHC	71	19 (26.76)	52 (73.24)			
Have you ever (even just once) altered or deviated from your medication regime due to cost i.e. halving medication dose etc?						
Total Sample	194	42 (21.65)	152 (78.35)			
LTHC	122	34 (27.87)	88 (72.13)	4.70	1.58, 16.6	.01
No LTHC	72	8 (11.11)	64 (88.89)			

*Note. As these additional analyses were exploratory and will be reflected on only in the sense of ascertaining whether the facet of currently pay prescription charges (vs. not) influenced the results, they were not included in the original Bonferroni p value correction. However, the Bonferroni correction adhered to in the main analyses ($p < 0.005$ (.05 / 9)) was applied when interpreting the results presented here.

Table S8. Qualitative summary of difference in statistical observations between the primary analysis (full sample) and exploratory analysis (people who currently pay for their prescriptions only).

Analysis	Difference observed*
Agreement with current per-prescription price	No difference in statistical observations. Proportion of participants disagreeing with current price is marginally elevated in people who currently pay only (56.35%) compared to full sample (53.2 %).
Consideration of per-prescription price prior to collection	No difference in statistical observations. Proportion of participants agreeing that they have previously considered the per-prescription price prior to collection is elevated in the people who currently pay only analysis (53.85%) compared to full sample analysis (41.3%)
Making financial sacrifices to afford prescriptions	No difference in statistical observations. Proportion of participants indicating they had not had to make financial sacrifices for their prescriptions was slightly lower in the people who currently pay only analysis (61.54%) compared to full sample (63.59%).
Not electing to collect prescription due to per-prescription charge	No difference in statistical observations. Fewer participants indicated they had never not collected a prescription due to the charge in the people who currently pay only analysis (68.39%) compared to full sample (76.61%).
Deviating from medication regime due to per-prescription charge	No difference in statistical observations. Fewer participants indicated they had never deviated from their medication regime due to prescription charges in the people who currently pay only analysis (77.95%) compared to full sample (83.33%).
Financial burden of prescription charges**	Difference in statistical observations. In the full sample analysis, participants with a long-term health conditions *were more likely* to agree that prescription charges place additional financial burden on themselves and/or their family. However, in the people who currently pay only analysis participants with a long-term health conditions *were not more likely* to agree that prescription charges place additional financial burden on themselves and/or their family.
Emotional burden of prescription charges	No difference in statistical observations. Participants with long-term health conditions were not more likely to agree that prescription charges placed additional emotional burden on themselves or their families in either analysis.

Note* Here we present a qualitative summary of the differences in the results obtained from the primary analysis compared to the secondary analysis. Please note no statistical analyses comparing the p values between the two analyses were conducted rather this is a qualitative synthesis.

**Denotes a difference in statical observations in full sample analysis compared to people who pay only analysis.

Table S9. Breakdown of long-term health conditions reported in the long-term health condition group.

Table S8. Breakdown of long-term health conditions reported.

LTHC (specific conditions included in category)	*N (%)*
Neurological Condition	130
Parkinson’s disease, hemiplegic migraine, essential tremor, transverse myelitis, Guillain-Barré syndrome, chronic pain, spinal stenosis, dystonia, stroke	
Cognitive Impairment	10
mild cognitive impairment, Alzheimer’s disease dementia, dementia	
Psychiatric Impairment	108
anxiety, depression, premenstrual dysphoric disorder, anorexia nervosa, obsessive compulsive disorder, bipolar disorder, post-traumatic stress disorder	
Rheumatic Illness	36
psoriatic arthritis, rheumatoid arthritis, osteoarthritis, gout	
Respiratory condition	24
asthma, chronic obstructive pulmonary disease, chronic bronchiectasis	
Metabolic condition	13
Type I and type II diabetes	
Cardiovascular condition	11
permanent atrial fibrillation, heart attack, heart failure, atherosclerotic heart disease, long QT syndrome, pulmonary embolus, sinus tachycardia, aortic valve and root replacement, postural orthostatic tachycardia syndrome	
Hypertension	47
Epilepsy	5
epilepsy, nocturnal epilepsy	
Autoimmune Disorder	19
Hashimoto’s, fibromyalgia, coeliac disease, hypergammaglobulinemia, Graves’ disease, undifferentiated connective tissue disease	

LTHC (specific conditions included in category)	*N (%)*
Blood disorder<br>human immunodeficiency viruses, anaemia, non-specified requiring splenectomy, immune thrombocytopenic purpura	10
Cystic Fibrosis	2
Cancers<br>Skin, breast, thyroid, bladder, prostate	7
Inherited conditions<br>Vohwinkel's disease, thalassaemia trait, Ehler-Danlos syndrome, hemochromatosis	5
Skin conditions<br>ichthyosis, eczema	2
Endocrine disorders<br>hypogonadism, hyperthyroidism, hypothyroidism	13
Gastrointestinal disorders<br>inflammatory bowl syndrome, colitis, Chron's disease	6
Reproductive disorders<br>polycystic ovaries, unspecified infertility, endometriosis	5
Neurodevelopmental disorder<br>attention deficit hyperactivity disorder	5

Number of LTHCs.	N (%)*
1	147 (55.06)
2	78 (29.21)
3	31 (11.61)
4	8 (3.00)
5	3 (1.12)

Note. Long term health condition (LTHC). As a substantial proportion of participants disclosed living with more than LTHC the total number of LTHC reported is greater than the total sample size and therefore cannot be calculated as a proportion of the total sample. Instead, we report the total number of LTHCs reported as a proportion of the total sample size. However, the number of LTHCs reported n does equate to total sample size therefore this value is also presented as a proportion.
*** Total sample size = 267**
