# Supplementary material for: Prescription charge policy acceptance among UK adults with and without long-term health conditions: a mixed-method survey
Source: BMJ Open. 2024 Sep 24;14(9):e085345. doi: 10.1136/bmjopen-2024-085345 (PMC11423742; doi:10.1136/bmjopen-2024-085345)
Supplement: Supplementary file 2 [file bmjopen-2024-085345.reviewer_comments.pdf]

PEER REVIEW HISTORY

BMJ Open publishes all reviews undertaken for accepted manuscripts. Reviewers are asked to complete a checklist review form and are provided with free text boxes to elaborate on their assessment. These free text comments are reproduced below.

ARTICLE DETAILS

**TITLE (PROVISIONAL)**	Prescription Charge Policy Acceptance Amongst UK Adults with and without Long-term Health Conditions: A Mixed-Methods Survey
**AUTHORS**	Readman, Megan Rose; Polden, Megan; Brighton, Lisa; Oluseye, Ayomide; Fairman, Ian; Parkinson, Ian; Parkinson, Caroline; Giebel, Clarissa

VERSION 1 - REVIEW

**REVIEWER NAME**	Varley, Aine
**REVIEWER AFFILIATION**	National University of Ireland Galway, Health Economics and Policy Analysis Centre
**REVIEWER CONFLICT OF INTEREST**	Currently employed by the Health Service Executive in Ireland. No competing interests to declare.
**DATE REVIEW RETURNED**	04-Mar-2024

**GENERAL COMMENTS**	Overall comments<br>I'd like to thank the authors for the opportunity to review this engaging and interesting piece of work.<br>I think this research adds an important perspective to the existing literature in this area, and would recommend acceptance for publication subject to revisions outlined here. Major comments relate to the Methods and Results sections.<br><br>Note: Comments and references to text location relate to pdf document titled "bmjopen-2024-085345_Proof_hi" (47 pages in total).

**REVIEWER NAME**	Kröger, Edeltraut
**REVIEWER AFFILIATION**	Centre integre universitaire de sante et de services sociaux de la Capitale-Nationale, Laval University Research center on primary care and services
**REVIEWER CONFLICT OF INTEREST**	i have no competing interests to declare.
**DATE REVIEW RETURNED**	06-Jun-2024

**GENERAL COMMENTS**	First of all, thanks to the authors for addressing a topic less studied in research on medication use, but nonetheless important: the role of cost for medication for chronic conditions. Given the rise in cost of living expenses in many countries around the world, this issue has become even more pressing.

	For me this manuscript addresses the larger research question under a mostly qualitative and UK centered angle. Research has to start somewhere and this is an important first step.<br><br>For the publication to become more meaningful to readers outside the UK, a short recapitulation on how prescription medication is paid for, and by whom, in the UK would be helpful, maybe even converting pounds into US dollars?<br><br>In the first pages, I noted some minor errors (see below). Regarding results and the discussion, a conceptual thread for the enumeration of results would be helpful, maybe a figure illustrating the characteristics of the sample of respondents and relating that to the types of reponses obtained.<br><br>For the discussion, an important question remains how the results relate to severity of the long term condition or other health expenses regarding it, to income of the respondants and finally to situations in other countries where legislation changes regarding medication cost are being considered (i.e. the Pharmacare project of the Canadian government.<br><br>Regarding ethics, please state how informed consent was obtained.<br><br>Regarding limitations, there needs to be more discussion on selection bias which always affects survey studies for which participants chose to participate in.<br><br>Finally, some minor points:<br>1. page 4, line 14 There are 2 points at the end of the sentence<br>line 46: prescription changes...or rather charges??<br>line 55: could one say "the current UK prescription charge policy<br>2. page 5: underline that study participation was entirely self determined, so that persons preoccupied with prescription charges were possibly more likely to participate<br>3. page 6, line 6: participant HAVE LTHC and not "are LTHC".<br><br>Also, the questions of the survey are included in the supplementary files - could the most important elements of it be clearly included in the main manuscript?

VERSION 1 – AUTHOR RESPONSE

Reviewer #1

1. Title Minor comment: I agree the quote ("I think it's bad enough…") captures the essence of the work quite well. However, I feel the title is too long overall. Ignore if broader consensus is that it's appropriate.

We appreciate your feedback regarding the length of the title of this manuscript. On reflection we agree that the title was lengthy and so have removed the quote from the title.

"Prescription Charge Policy Acceptance Amongst UK Adults with and without Long-term Health Conditions: A Mixed-Methods Survey" (pg. 1)

2. Abstract, Strengths and Limitations Minor comment: P4 L30-32 “with themes including…” Editors will clarify, but seems this missing a colon and subsequent numbered elements should not be capitalised.

Apologies for these grammatical errors, this has now been corrected.

“. Qualitative findings indicate perceived inequalities in current policy with themes including: (1) the need for re-evaluation; (2) the burden of prescription charges; (3) inconsistencies and inequalities in current policy; and (4) positive reflections of prescription charge policy.” (Pg. 2)

3. Introduction Minor comments: P6 L7-8 “…with the latest charge of £9.65 per prescription being brought into effect in April 2023.” It might be worth clarifying here that this relates to a prescription regardless of the number of items contained (if I’ve understood correctly). In other jurisdictions, prescriptions charges can be applied per item (e.g. Ireland) – it may be confusing for readers unfamiliar with the NHS.

Thank you for highlighting the need for further clarification here. As of April 2024 the prescription charge is £9.90 per prescribed item, that is if there are 3 items on the prescription it will come at a cost of £29.70. Therefore, we have amended the manuscript to clarify this, you will also note that we have updated the costing to reflect the 2024 increase.

“Excluding the period between 1965 and 1968 when prescription charges were abolished in the UK, the price per prescribed item has continually risen, with the latest charge of £9.90 per prescribed item being brought into effect in April 2024 [1].” (Pg. 4)

4. Introduction Minor comments: P6 L14 “specific medical conditions (MedEx)..” Referred to a ‘MedEx criteria’ in nearly every other mention in the manuscript. Suggest revising to ensure consistency, as confusing otherwise for those unfamiliar with system.

We thank you for highlighting the confusion this may cause to the reader less familiar with this system. We have amended the first reference to MedEx to be MedEx Criteria to alleviate this confusion.

“….4) people living with specific medical conditions (MedEx criteria).” (Pg. 4)

5. Introduction Minor comments: P6 L29-30 “…the Department of Health and Social Care introduced a Prescription Prepayment Certificate (PPC).” Additional detail on the timelines for PPCs (e.g. year first introduced) might be helpful here.

We thank you for highlighting this. The corresponding author has contacted the NHS Business services (who manage the PPC), the NHS England health inequalities improvement programme team, the prescription charge coalition lead and several practicing pharmacists. Unfortunately none of the above individuals are aware of the date of the PPC initiative was introduced. Moreover, the date of initiative initiation is not clearly documented on the government achieves. As such, we have refrained from adding such information.

6. Introduction Minor comments: P6 L33 It's unclear to most readers from this what the difference between "multiple and repeat prescriptions" would be. Suggest clarification.

We thank you for highlighting the lack of clarity here the manuscript has been updated to clarify this matter.

"PPC's specifically aim to reduce the costs of prescriptions for people who repeatedly require multiple prescribed items , with the PPC minimising prescription costs for people who buy more than 3 prescribed items in 3 months or 12 prescribed items in 12 months." (pg.4).

7. Introduction Minor comments: P6 L43 "…with over a third of respondents (31%) citing…" 31% is not 'over a third' (33.33333%). Suggest revision.

We apologise for the incorrectness of this statement this has now been updated.

"Furthermore, awareness of the PPC appears to be driven by word of mouth with 31% of respondents citing the source of awareness being friends/family."(Pg. 4)

8. Introduction Minor comments:2014 Prescription Charges Coalition survey is capitalised (P6 L49-50), but 2023 prescription charge coalition survey (P6 L37 and P7 L8) are not. For consistency, suggest capitalising all (and any other instances where this is referenced that I haven't listed here).

We thank you for highlighting this inconsistency. The authors have subsequently checked the entire manuscript and amended any capitalisation inconsistencies.

9. Methods: Major comment: I think the methods section would benefit from a more structured approach.

Upon reflection we appreciate that the methods may have been somewhat unstructured. We have amended the sequential presentation of the methods to follow the APA 7th Edition guidelines (Participants Materials procedure) and ensured that all information is presented under the appropriate heading. Moreover, within the materials section we have introduced an APA 7th Edition level heading structure.

10. Methods: Major comment: Additional information on how the survey was developed would be welcome, given this instrument is not validated and has been created specifically for this study. For example, how were the items/questions selected for inclusion (e.g. to align with any particular framework or themes)? What was the role of patient experts in developing the survey? Who proofed the survey (i.e. was language appropriate for target audience)? Was it piloted? If so, were any changes made on the basis of the pilot?

Further information regarding the development of the survey and the role of patient experts (public advisors) has been embedded within the manuscript.

Survey Development

"The survey was co-developed with the project public advisory team which consisted of two people living with LTHCs (IF and CP) and family member of a person living with a LTHC (IP). Co-development occurred following a five steps process: 1) The Prescription Charge Coalition 2023

survey was consulted and the gaps in this survey assessed (MR and MP), 2) a skeleton survey was developed based on the gap identified in the Prescription Charge Coalition 2023 survey and existing literature (MR and MP), 3) the wider research team provided feedback on the skeleton survey and it was revised accordingly, 4) public advisors provided feedback on the skeleton survey, particularly focusing on whether they feel it appropriate addressed their lived experience, and it was revised accordingly, 5) the amended survey was redistributed to public advisors for assessment of readability and accessibility. The resulting survey was then piloted on 7 participants (3 people with LTHCs and 4 people without LTHCs). The pilot revealed that the survey was appropriate in terms of readability, length and content and so was finalised." (Pg. 5-6)

11. Methods Minor comment: P7 L15 The OSF link indicates further access/permission required on that site. This could be an error on my part, but might be worth clarifying if change in access status etc. is required.

We apologise for this formatting error the URL link was meant to lead to an anonymised version of the OSF registration page. The URL has now been updated and checked by several people independent of the research team. Please do let me know if this is still inaccessible.

"(OSF; https://shorturl.at/IrvnS)"(pg. 5)

12. Methods Minor comment: P7 L15-16 "The only small deviation…" Suggest 'minor' would be more appropriate language than 'small'.

This amendment has been implemented

13. Methods Minor comment: P7 L47 "…aged 14+…" Should indicate years. Also, ≥18 years (P7 L28) and <14 (P7 L48) used elsewhere. Suggest using ≥/>/< etc. rather than + for consistency.

We have amended this to clearly denote 14> years, and have also updated the remaining manuscript for consistency

"aged 14> years, and the number of people residing in the same household aged <14 years" (Pg. 6).

14. Methods Minor comment: P P8 L5-6 "…any of twelve pre-set LTHCs…"Was any particular classification system or framework used to select these LTHCs, or was this list generated by the authors independently? Suggest describing and justifying approach (e.g. comparability with existing research).

Further information as to how the pre-set LTHCs were selected has been embedded within the survey measures section of the methods.

"The pre-set list of LTHCs was developed based on the pre-specified conditions included within the Prescription Charges Coalition 2023 survey [4] and was updated to ensure both and the National Health Service (NHS) Quality and Outcomes Framework 20 common conditions [9] and UK Department of Health and Social Care Major Conditions Strategy major condition groups [10] were included. The inclusion of the other non-specified group further ensured that people who identified with all LTHCs were included."(Pg. 6).

15. Methods Minor comment: Separately, the authors have been quite inconsistent in their convention for numbering. Suggest picking a rule and ensuring consistent application throughout the manuscript (e.g. words for zero through nine and numerals from 10 onwards).

The manuscript has been amended to ensure words are used for zero through nine and numerals afterward.

16. Methods Minor comment: P8 L13-14 "…the costs associated with these medications." Suggest clarifying these were monthly costs (as indicated in the supplementary material).

We apologise for this lack of clarity the manuscript has been amended to reflect this

"… and the monthly costs associated with these medications"(Pg. 6)

17. Methods Minor comment P8 L17-20 & Supplementary Material 1, Table 4 Please clarify if you included any additional descriptor or information regarding the PPC to aid the respondent in recognising what the scheme is? I would have concerns that, depending on the level of health literacy of the respondent, the name of the scheme alone might not have been sufficient.

Indeed, in attempt to mitigate the influence of different phraseology and health literacy participants were provided with further clarification as to what the PPC initiative is. This has now been clarified in the manuscript.

"To ensure that differences in the terminology used to refer to the PPC did not impact participants responses, this question was accompanied with a short description of what the PPC ("A certificate that covers all your NHS prescriptions for a set price, like a prescription charge season ticket") is and a link to the NHS website for further clarification of what the PPC initiative is." (Pg. 6)

18. Results Major comment: The results section requires considerable editorial revision. The current draft is very much focused on narrative presentation of results, and is consequently very long and difficult to read. Suggest presenting the quantitative results in tabular form to ensure completeness of reporting, and limiting narrative description to key findings.

We appreciate that the result section of the initial manuscript was quite lengthy and hence difficult to follow. As per your suggestion we have presented the quantitative results in tabular form and condensed the narrative description. Due to the format of the outcome variables (i.e. binary or multi-level variables) we conducted both multinomial and binary logistic regression. For clarity we have presented the quantitative results in two separate tables organised by the type of analysis conducted, the order of result presentation within the table follows the sequential order in the results section.

19. Results Major comment: Please ensure that the results are aligned with the methods section (and protocol), and are presented in the same order between sections.

We apologise for the lack of consistency in the organisation of the method and results, this has been amended to ensure consistency.

20. Results Major comment: For clarity, would also suggest amalgamating supplementary materials 1 and 2 and ensuring they are presented in a clear and consistent manner. For example, title tables in the main manuscript as Table X, and title tables in the supplementary material as Table SX. As it currently stands, there two tables in the Supplementary Materials 2 file titled Table S1 and Table 1. There is also another Table 1 in the main manuscript, and another Table 1 in Supplementary Materials 1. All of these should be reviewed to ensure there is no ambiguity. Consider subdivision as appendices within the one supplementary document if authors wish to distinguish between protocol and supplementary results.

As per your suggestion supplementary materials 1 and 2 have now been amalgamated and have been proofed to ensure clear and consistent reporting. The newly amalgamated supplementary materials have been subdivided and a clear contents has been embedded to facilitate easy reading. Please note, in line with your prior suggestion to tabulate quantitative findings the additional exploratory findings have been updated and are now presented in tabular form.

21. Results Major comment: I cannot access the data via the Open Science Framework link to verify the results reported here.

We apologise for this issue. This should now be resolved and the data should be accessible via the below link.

“(OSF; https://shorturl.at/lrvnS)”(pg. 5)

22. Results Minor comment: P9 L4-30 (‘Sample characteristics’) This section is too long. Consider revising. In many instances, results report only % (and not corresponding n). This can be confusing and it’s not always clear which denominator is used.

This section has now been condensed and the corresponding n has been embedded in instances where % are reported.

“381 people aged 19-84 (M= 47.29 (17.81)) participated in this survey: 267 people (70%) who reported having a LTHC and 114 people (30%) who did not report having a long-term health condition. Across the full sample, participants were predominantly female (n = 256, 67.19%) from a white ethnic background (n= 358, 93.96%), educated up to degree level or equivalent (n = 277, 72.70%), and in work (working full (n =163, 42.78%), or part time (n = 62, 16.27%)). Participants spread all IMD quintiles and homes of residence spanned most of England (See figure 2 for the local authority geographical regions represented; See Table 1 for full sample characteristic breakdown).

Participants who reported having a LTHC tended to be older, were more likely to be working full time, and were from more deprived IMD localities. Neurological conditions, psychiatric impairments, and hypertension were the most common LTHCs reported (See supplementary material; Table S6 for full breakdown of LTHCs reported). The majority of participants, who reported having a LTHC, reported having one condition (n = 147, 55.06%). However, a substantial proportion of participants reported living with several LTHCs (2 conditions, n = 78, 29.21%), 3 conditions, n = 31, 11.61%)). A small proportion (n = 26 , 9.74%) of participants with a LTHC indicated that they were not currently taking any long-term medications. These participants disclosed living with LTHCs for which non-pharmacological therapies are often first line treatment.” (Pg. 7-8).

23. Results Minor comment : P9 L4 I missed where the authors described how participation was defined. Did respondents have to complete all items in the survey to be processed as respondents? How did the survey software handle those who started but did not conclude the survey? Would it allow survey completion without responding to all items? If participation is limited to those who responded to every item only (i.e. you should expect n=381 responses to every item), it may be reasonable to mention this somewhere in the manuscript.

We appreciate that a clear description of how missing data was dealt with was lacking from the main manuscript (however please note this was detailed in the protocol). Therefore, we have embedded a brief summary of the treatment of missing data within the data analysis section of the methods.

"To maintain participants right to not provide data for a given question the survey was designed in a way which enabled response submission with incomplete answers. Therefore, for all analyses the entire sample who have data available for the given outcome variable will be analysed. To maximise the quantity of data available participants with missing data, and participants who responded "prefer not to say", will be excluded from the analysis for which they have missing data only." (Pg. 7)

24. Results Minor comment: P9 L4-8"Three hundred and eighty-one people participated in this survey: 267 people (70%) who reported having a LTHC and 114 people (30%) who did not report having a long-term health condition. Participants were predominantly female (67.2%) from a white ethnic background (94%), educated up to degree level or equivalent (72.7%),"

Underlined: see previous comment regarding inconsistency in convention for numbering. I appreciate this was probably motivated by not wanting to open a sentence with a number, but it is distracting when both words and numerals are used in the same sentence.

Bold: suggest consistency in number of decimal places used for percentages.

You are indeed correct in assuming we did not want to commence a sentence with a number and, therefore wrote it out in full. However, we appreciate your comment regarding consistency and so have updated this to read 381 people participated. We also apologise for the prior lack of consistency in the decimal place reporting, the entire manuscript has been checked and updated so all value are presented to 2dp with the exception of p values between 0.01 and 0.001 which are reported to 3dp (As per APA recommendations).

"381 people aged 19-84 (M= 47.29 (17.81)) participated in this survey: 267 people (70%) who reported having a LTHC and 114 people (30%) who did not report having a long-term health condition. Across the full sample, participants were predominantly female (n = 256, 67.19%) from a white ethnic background (n= 358, 93.96%), educated up to degree level or equivalent (n = 277, 72.70%), and in work (working full (n =163, 42.78%), or part time (n = 62, 16.27%)). Participants spread all IMD quintiles and homes of residence spanned most of England (See figure 2 for the local authority geographical regions represented; See Table 1 for full sample characteristic breakdown)." (Pg. 7).

25. Results Minor comment: P9 L17-18 "The majority of participants, who reported having a LTHC, reported having one condition (n = 147)." Given the sentence states "the majority", it would be useful to indicate what n=147 is as a proportion of the appropriate denominator. Whether the full study population or population with LTHCs is considered as the denominator is the authors' decision – either way, it should be clear to the reader which population it pertains to.

We have now included the proportion of participants with a LTHC who reported having one, two and three condition(s) respectively in the main body of the manuscript. These proportions and the respective denominator are also clearly detailed in Table 1.

“Most participants, who reported having a LTHC, reported having one condition (n = 147, 55.06%). However, a substantial proportion of participants reported living with several LTHCs (2 conditions, n = 78, 29.21%), 3 conditions, n = 31, 11.61%)).” (Pg. 8).

26. Results Minor comment: P9 L22 “A small proportion (9.74%)…” Suggest a minority, rather than small. Suggest ensuring consistency in number of decimal places used for percentages (as above).

This phraseology has been amended as per your recommendation. Moreover, the entire manuscript has been checked and updated so all value are presented to 2dp with the exception of p values between 0.01 and 0.001 which are reported to 3dp (As per APA recommendations).

“A minority (n = 26, 9.74%) of participants with a LTHC indicated that they were not currently taking any long-term medications. These participants disclosed living with LTHCs for which non-pharmacological therapies are often first line treatment.” (Pg. 8).

27. Results Minor comment: P9 L29 Please clarify if appropriate term is “vohwinkels disease” or Vohwinkel Syndrome and update the manuscript if required.

We agree that the most accepted terminology is Vohwinkel Syndrome and so have updated the manuscript with respect to this. Please note the reference to Vohwinkel syndrome on Pg. 9 has been removed as per your prior comment to condense this section, however, reference is still made to Vohwinkel Syndrome in the Appendix.

28. Results Minor comment: P9 L33 to P10 L36 All quantitative results here should be presented somewhere in tabular form. This would help to cut down on the amount of narrative reporting required.

As per your prior comment (18), we have presented the quantitative results in tabular form and condensed the narrative description. Due to the format of the outcome variables (i.e. binary or multi-level variables) we conducted both multinomial and binary logistic regression. For clarity we have presented the quantitative results in two separate tables organised by the type of analysis conducted, the order of result presentation within the table follows the sequential order in the results section.

29. Results Minor comment: Please ensure all abbreviations are defined on first use (e.g. RRR, OR, GP etc.).

We have now embedded a brief description of the statistical abbreviations used within the data analysis section. Moreover we have ensured all other abbreviations are defined on first use.

“Multinomial logistic regression results are presented as relative risk ratio (RRR) along with 95% confidence intervals (CI) and p-value. Binary logistic regression results are presented as odds ratios (OR) along with 95% confidence intervals (CI) and p-value.” (Pg. 7).

30. Discussion & Conclusion: Generally, the discussion section felt quite long and repetitive, and may benefit from being streamlined. I found it difficult to review this section in full without complete quantitative results and would like the opportunity to re-review when these are provided.

We have carefully considered the clarity and conciseness of the discussion and conclusions and made amendments where we found the content to be repetitive. As per prior comments the results have now been presented in tabular form. Therefore, we welcome any additional comments that may arise on reflection of this. Please note as amendments have been made to the entire discussion section we do not present the amendments here.

31. Discussion & Conclusion: Minor comment P12 L21-22'However' used twice in close succession – difficult to read.

We thank you for highlighting this, we have removed the second 'however'.

32. Discussion & Conclusion: Minor comment P12 L40-60

Medication non-adherence is a very nuanced issue and influenced by a broad range of factors. I don't think that is appropriately acknowledged here, and the implicit assumption that reducing prescription costs will improve adherence is somewhat optimistic. This paragraph is also too long.

We thank you for highlighting this important facet. We have amended this discussion to acknowledge the influence of additional factors that may influence medication adherence and place greater caution on the assumptions drawn. Moreover, the length of this discussion has been reduced.

"Prior research, although primarily non-UK based, has shown that medication costs may be significant barriers to medication adherence [4, 14]. Congruent with these observations 23.71% of participants reported not collecting a prescritpion and 16.80% of participants reported having deviated from their prescribed medication regime (e.g. skipping doses or half-dosing) due to the associated per-prescribed item costs. Medication non-adherence can lead to reductions in functional abilities and quality of life, the occurrence of additional health complication, premature death, and increased use of medical resources (e.g. hospitalisation) [15, 16, 17]. Thus, per-prescribed item cost may to some extent influence the likelihood of an individual experiencing poorer health outcomes. It is, however, important to note that a wide range of factors, beyond per-prescribed item charges, influence medication adherence [18]. Thus, these assumptions should be treated with a degree of caution. Furthermore, whilst per-prescribed item costs aim to produce income for the NHS, non-adherence behaviours may ultimately result in greater costs to the NHS. Indeed, it has been estimated that medication non-adherence in asthma, type 2 diabetes, high cholesterol/coronary heart disease, hypertension and schizophrenia may cost the NHS up to £930 million per annum [19], and ~4.60% of total global health expenditures could be avoided with better medication adherence [20]."(Pg. 11)

33. Discussion & Conclusion: Minor comment P13 L54 to P14 L21 The limitations start with 'First…' (P13 L56) and finish with 'Finally…' (P14 L16), but it's difficult to delineate between various limitation between these sentences. Use of 'Second…', 'Third…' etc. as appropriate might help with this.

We have amended this paragraph to aid in the delineation of the various limitations.

34. Table 1 P18-19 Please include abbreviations in the footnotes. Suggest ensuring consistency in number of decimal places used. Age is missing unit of measurements. Suggest indicating in footnotes what IMD levels indicate, as it will not be immediately apparent to those not familiar with UK.

Definitions for all abbreviations have added to the footnotes. As per prior comments Table 1 has been updated such that all values are to 2dp (with the exception of P values <.01). The unit of measurement, years, for age has been added. A brief description what IMD values are and what the levels indicate has been embedded in the footnote.

"*** IMD is an index of neighbourhood deprivation generating one deprivation score for income, employment, education, health, crime, barriers to housing and services, and living environment. In accordance with the Ministry of Housing, Communities & Local Government English indices of deprivation 2019, quintile '1' indicates the most deprived neighbourhoods, and quintile '5' indicating the least deprived neighbourhoods [8]. Not all participants provided postcode data, therefore, IMD could not be calculated for all participants. The percentages computed for the IMD variable are true percentages of the participants for which an IMD value could be calculated." (Pg. 17).

35. Table 2 P20-22 Please include abbreviations in the footnotes.

Definitions for all used abbreviations have been added to the footnotes.

36. Supplementary Materials 1 General Some tables are titled Table X, whereas others are titled Table SX. Please address in line with previous major comment under Results.

As per your suggestion supplementary materials 1 and 2 have now been amalgamated and all table titles have been updated accordingly. Specifically, tables within the supplementary materials are titled Table S1-S9 and the 4 tables in the main manuscript are titled Table 1-4.

37. Supplementary Materials 2 P26-27 Table S1 Suggest including denominator in header. Whether the full study population or population with LTHCs is considered as the denominator is the authors' decision. Suggest including footnote to clarify if required. Please include abbreviations in the footnotes.

There are multiple spelling mistakes (e.g. Chron's, Heamochromotosis [sp.]). Please review all terms used and ensure all are correct before resubmitting. Inconsistent use of capitalisation when listing conditions per category; suggest picking one option and sticking with it. My preference would be to limit capitalisation only where it's indicated (e.g. Alzheimer's).

In the first half of this table (now table S9) we report the number (n) of participants who disclosed living with a specific long term health condition. Given that several participants disclosed living with more than one long term health condition the total number of long term health conditions reported is greater than the total sample size and therefore cannot be calculated as a proportion of the total sample size. Thus, we have elected not to report proportion here and have reported the raw n. This has been justified in the footnote of the table. We have also defined all abbreviations in the footnote.

We have checked all spellings and capitalisation within Table S9.

38. Supplementary Materials 2 P27 L54-P28 L38 Narrative results pertaining to ‘Acceptability and perceptions of prescription charge policy’ should be presented in tabular form.

As per your prior suggestion the exploratory analyses have been reformatted into tabular form (following the exact format of the primary analysis).

39. Supplementary Materials 2 Quantitative results should be presented quantitatively in tabular form. Reporting ‘no difference in statistical observations’ is not sufficient. Report numbers, proportions, p-values and indicate statistical test used to make comparison for each test. If authors feel additional narrative description is indicated, limit to key findings.

This table aimed facilitate easily comparison of the primary analysis and exploratory analysis findings. To do so we qualitatively summarised the differences in the results obtained from the two analyses. That is, no statistical analyses comparing the p values between the two analyses were conducted. This has been clarified and clearly detailed in the footnotes of this table in the supplementary materials (Table S7). However, as per comment 38 we have also reformatted the exploratory analysis and presented them in tabular format with the p values and associated statistics clearly detailed.

Reviewer #2

1. For the publication to become more meaningful to readers outside the UK, a short recapitulation on how prescription medication is paid for, and by whom, in the UK would be helpful, maybe even converting pounds into US dollars?

We thank you for highlighting the need for further clarification to ensure the manuscript is accessible to researchers outside of the UK. We have embedded a short recapitulation on prescription charges in the UK in the introduction. We have refrained from converting the fees paid to dollars as the prescription charge system employed in the US is vastly different. We have, however, embedded a discussion regarding how these findings may inform policy internationally in the discussion (please refer to point 4 below).

“Prescription charges, an upfront monetary fee associated with prescribed medications, were first introduced in the UK in 1952 [1]. Excluding the period between 1965 and 1968 when prescription charges were abolished in the UK, the price per prescribed item has continually risen, with the latest charge of £9.90 per prescribed item being brought into effect in April 2024 [1]. In 1968, a system of exemptions from prescription charges was introduced. This system enables people meeting the following criteria to be exempt from paying per prescribed item charges; 1) people in defined age groups (people under the age of 16, people aged 16-18 in full time education, and people over the age of 60), 2) people who are pregnant or have had a baby in the last 12 months, 3) people who receive certain state benefits, and 4) people living with specific medical conditions (MedEx criteria). Subsequently, people aged 18-60, who do not receive state benefits, are not pregnant or have had a baby in the last 12 months, and are not living with medical condition covered by the MedEx criteria, are required to pay an upfront fee of £9.90 per prescribed medication on collection. For the financial period of 2021-2022 it was estimated that £652 million revenue was collected from prescription charges in the UK [2]. “ (Pg. 2)

2. In the first pages, I noted some minor errors (see below)

a. page 4, line 14 There are 2 points at the end of the sentence

We thank you for pointing this out, this has been amended.

b. line 46: prescription changes...or rather charges??

We thank you for pointing this out, this has been amended.

c. line 55: could one say "the current UK prescription charge policy

We thank you for pointing this out, this has been amended.

d. page 5: underline that study participation was entirely self determined, so that persons preoccupied with prescription charges were possibly more likely to participate

We thank you for this suggestion. We have embedded a brief statement regarding self-selection in the Participants section of the Methods. Moreover, we have further discussed the potential influence of self-selection on the subsequent findings in the discussion.

"Participants were recruited via social media advertisements, including adverts issued by Parkinson's UK, and email adverts to local research networks. Thus, participation was self-selected." (Pg. 5).

"Second, as this study recruited a volunteer sample the issue of self-selection bias must not be overlooked. It is not unreasonable to assume that several participants elected to partake in this survey due to an individual preoccupation with medication charges. Thus, the findings obtained here may not be representative of people to whom prescription medications are less of a concern. Whilst we attempted to in part mitigate this issue by recruiting people who do not have LTHC further studies should seek to recruit a larger sample with a particular focus on people who do not frequently require prescription medications." (Pg. 12).

e. page 6, line 6: participant HAVE LTHC and not "are LTHC".

We thank you for pointing this out, this has been amended.

3. Regarding results and the discussion, a conceptual thread for the enumeration of results would be helpful, maybe a figure illustrating the characteristics of the sample of respondents and relating that to the types of reponses obtained.

We thank you for highlighting that a figure may aid the comprehensibility of the results. As per the suggestions of reviewer 1 all quantitative findings have been presented in tabular form. Thus, to

prevent the overuse of tables and figures we have elected not to embed any further figures. However, if the tables presented do not clearly address your concern we are willing to reconsider this suggestion.

4. For the discussion, an important question remains how the results relate to severity of the long term condition or other health expenses regarding it, to income of the respondents and finally to situations in other countries where legislation changes regarding medication cost are being considered (i.e. the Pharmacare project of the Canadian government.

The authors agree that these are very important aspects to discuss and hence have amended the discussion to embed these considerations. However, please note to ensure the manuscript does not become too lengthy these discussion are concise.

5. Regarding ethics, please state how informed consent was obtained.

Further information regarding obtaining informed consent has been embedded within the methods section.

"On following the online survey link participants were directed to the study information sheet clearly detailing the aims of the study and associated task. If participants elected to proceeded, they then provided digitalised informed consent and completed a reCAPTCHA identification check to ensure no bots completed the study. On attainment of informed consent participants were presented the main survey." (Pg. 7).

6. Regarding limitations, there needs to be more discussion on selection bias which always affects survey studies for which participants chose to participate in.

We thank you for highlighting the need to acknowledge this relevant issue. A brief discussion regarding self-selection bias has been embedded within the limitations section of the discussion.

"Second, as this study recruited a volunteer sample the issue of self-selection bias must not be overlooked. It is not unreasonable to assume that several participants elected to partake in this survey due to an individual preoccupation with medication charges. Thus, the findings obtained here may not be representative of people to whom prescription medications are less of a concern. Whilst we attempted to in part mitigate this issue by recruiting people who do not have LTHC further studies should seek to recruit a larger sample with a particular focus on people who do not frequently require prescription medications." (Pg. 12).

7. Also, the questions of the survey are included in the supplementary files - could the most important elements of it be clearly included in the main manuscript?

We thank you for this suggestion the core questions relating to the acceptability and impact of per-item prescription charges have now been embedded within the main body of the manuscript.

VERSION 2 - REVIEW

**REVIEWER NAME**	Kröger, Edeltraut
**REVIEWER AFFILIATION**	Centre integre universitaire de sante et de services sociaux de la Capitale-Nationale, Laval University Research center on primary care and services
**REVIEWER CONFLICT OF INTEREST**	I have no competing interests with regard to this manuscript.
**DATE REVIEW RETURNED**	16-Aug-2024

**GENERAL COMMENTS**	The revised version addresses some of the suggestions made on the first version. I could not find a point by point answer to comments - I don't know whether this is due to BMJ Open's review progress. Some points remain to be addressed:<br>1) There is no mention on ethical concerns: were the survey participants aware that their responses would be used in the publication of the research results?<br>2) Some of the tables do not repeat headers on each place which makes them harder to read.<br>3)The main results are presented in the results section and discussed. I have a problem with the fact that the presence of LTHC did not seem to impact the recovery of prescriptions by patients bu that LTHC patients declared less adherence to taking prescribed medications. As presented, this latter difference between patients with and without LTHC is not explained. The reader might think that this difference is due to prescription charges, but it might also be due to the complexity of medication regimes for patients wiht LTHC or to adverse effects of these numerous medications. This needs to be discussed, There are two important points to prescription medication charges: 1) do they make life significantly harder for those whose life is already harder (i.e. LTHC patients)? If yes, what can be done about this...in England? 2) Do the fees prevent LTHC patients from adhering to therapeutic regimens and thus increasing their risk of further deterioration of their LTHC and (costly) consequences? I think the discussion could make the salient results and their implications a bit clearer. The manuscript presents a lot of material and the reader may lose the main thread of the study if there in"t a bit more coherence of conclusive results in the end.<br>4) Just a very minor point: "Current MedEx criteria is outdated" should be "...are outdated".

VERSION 2 – AUTHOR RESPONSE

Reviewer #2

1. The revised version addresses some of the suggestions made on the first version. I could not find a point by point answer to comments - I don't know whether this is due to BMJ Open's review progress.

We apologise that you were not able to view the point by point response that was uploaded in response to the prior round of revisions. We are not sure why this has occurred.

2. There is no mention on ethical concerns: were the survey participants aware that their responses would be used in the publication of the research results?

We thank you for highlighting further information regarding ethical considerations would be of benefit. We have embedded a brief statement regarding such matters in the main manuscript.

"If participants elected to proceeded, they then provided digitalised informed consent and completed a reCAPTCHA identification check to ensure no bots completed the study. On attainment of informed consent participants were presented the main survey. All participants provided consent for their anonymised date to be included in the present publication and other outputs relating to this study." (pg. 7)

3. Some of the tables do not repeat headers on each place which makes them harder to read.

We thank you for highlighting that the lack of header repetition may impede the readability of the tables. We have subsequently updated all tables (both in the main manuscript and supplementary materials) to ensure that the headers are repeated should they span more than one table. Please note due to the length of the tables we have not included them here.

4. The main results are presented in the results section and discussed. I have a problem with the fact that the presence of LTHC did not seem to impact the recovery of prescriptions by patients but that LTHC patients declared less adherence to taking prescribed medications. As presented, this latter difference between patients with and without LTHC is not explained. The reader might think that this difference is due to prescription charges, but it might also be due to the complexity of medication regimes for patients with LTHC or to adverse effects of these numerous medications. This needs to be discussed.

We thank you for highlighting this interesting point and acknowledge that this needs to be reflected upon in the manuscript. Therefore, we have embedded a brief discussing regarding this point in the discussion.

“It is, however, important to reflect on the differences observed between people with and without LTHCs with respect to prescription collection and medication regime adherence. Specifically, although people with LTHCs were more likely to deviate from their medication regime, we did not observe a significant difference in the occurrence of prescription non-collection between people with and without LTHCs. Medication regimes for people living with LTHCs tend to be more complex and lengthy. Thus, it may be that the non-adherence with medication regimes reported here was in part due to the complexity of the medication regime as opposed to the cost associated with the prescription. Indeed, prior evidence has shown that medication regime complexity negatively impacts the level of medication adherence in people with multiple conditions [22]. However, the question posed to participants in the present study was “Have you ever (even just once) altered or deviated from your medication regime due to cost”. As the question posed specifically asked participants to reflect on the impact of cost this assumption may be unlikely.” (Pg. 11-12).

5. There are two important points to prescription medication charges: 1) do they make life significantly harder for those whose life is already harder (i.e. LTHC patients)? If yes, what can be done about this...in England? 2) Do the fees prevent LTHC patients from adhering to therapeutic regimens and thus increasing their risk of further deterioration of their LTHC and (costly) consequences? I think the discussion could make the salient results and their implications a bit clearer. The manuscript presents a lot of material and the reader may lose the main thread of the study if there isn’t a bit more coherence of conclusive results in the end.

We thank you for highlighting these important questions. We have embedded further reflection on these points throughout the discussion. Moreover, we have reviewed the discussion and made edits to bring the salient results and their implications to the forefront. Please note the amendments made were sporadic throughout the entire discussion, therefore, we do not highlight them here.

6. Just a very minor point: "Current MedEx criteria is outdated" should be "...are outdated".

This grammatical error has now been rectified.

VERSION 3 - REVIEW

**REVIEWER NAME**	Kröger, Edeltraut
**REVIEWER AFFILIATION**	Centre integre universitaire de sante et de services sociaux de la Capitale-Nationale, Laval University Research center on primary care and services
**REVIEWER CONFLICT OF INTEREST**	I have no competing interests regarding the material presented in this manuscript.
**DATE REVIEW RETURNED**	05-Sep-2024

**GENERAL COMMENTS**	Thank ou very much for addressing my comments and concerns - I think this research will add important points to clarify the ongoing

	discussion of the cost of and the payment for long term prescription medication.
